# Participatory development of strategies to combat congenital syphilis: action research^*^

**DOI:** 10.1590/1518-8345.8014.4894

**Published:** 2026-08-10

**Authors:** Juliana Sanches Ravagnani, Juliane Andrade, Lucas Cardoso dos Santos, Rúbia Aguiar Alencar

**Affiliations:** 1 Universidade Estadual Paulista, Faculdade de Medicina de Botucatu, Botucatu, SP, Brazil.; 2 Prefeitura de Lins, Secretaria Municipal de Saúde, Lins, SP, Brazil.; 3 Universidade de São Paulo, Escola de Enfermagem, São Paulo, SP, Brazil.

**Keywords:** Congenital Syphilis, Delivery of Health Care, Health Services Research, Health Planning, Patient Care Bundles, Primary Health Care.

## Abstract

**Objective:**

to describe the development of a strategic action plan to combat congenital syphilis, carried out in a participative manner with professionals from the Health Care Network.

**Method:**

qualitative research, using an action research approach based on Freire’s framework. Conducted in a municipality in the interior of São Paulo, it involved 56 health professionals. The absence of local strategies and epidemiological data led to the holding of a workshop in three meetings. The activities were recorded on murals, written evaluations, and field diaries, and analyzed using Content Analysis.

**Results:**

the meetings promoted the involvement of professionals, revealed weaknesses in the management of syphilis, and prompted the formulation of proposals, such as restructuring testing and diagnosis flows, timely prescription and treatment, active search, monitoring tests, treatment of partners, and adequate management of syphilis cases in pregnant women. Three categories emerged, and as a final product, a strategic action plan was developed to address congenital syphilis with practical applicability in the network’s services.

**Conclusion:**

the collaborative process strengthened professionals’ autonomy and co-responsibility, integrating technical knowledge and local experience. The plan has been implemented and has contributed to improving the response to congenital syphilis within the Brazilian Unified Health System.

## Highlights

**(1)** Strategic action plan to combat congenital syphilis. **(2)** Action research as a methodological approach that enables participatory construction. **(3)** Participatory and integrated strategies strengthen the fight against syphilis. **(4)** Importance of cooperation and systematization of networking. **(5)** Feasible, achievable, and affordable network response to congenital syphilis.

## Introduction

Syphilis is a sexually transmitted bacterial infection that is preventable and curable, and has become a growing concern in terms of global public health. The World Health Organization (WHO) estimates that the number of cases has increased significantly in recent years, with significant variations across regions of the world^1^ ^-^ ^3^. 

The Americas, with disparities between North America (the highest incidence), Latin America, and the Caribbean, account for the highest global burden of the disease, with approximately 3.37 million new cases, corresponding to 42.0% of recent infections, followed by regions in Africa and Southeast Asia^1^ ^,^ ^3^ ^-^ ^4^. This trend directly reflects the increase in cases of congenital syphilis (CS), with approximately 68,000 live births affected per year worldwide^1^ ^,^ ^3^ ^,^ ^5^, with a broad spectrum of clinical manifestations and adverse outcomes such as spontaneous abortion, fetal death, prematurity, low birth weight, congenital anomalies, and neurological sequelae^5^ ^-^ ^8^.

In this context, addressing CS requires favorable conditions for developing actions and strategies to improve prenatal and postpartum health care. Among these, the implementation of public health policies, the commitment of managers, teamwork and networking, as well as adequate prenatal care with early detection and linkage to services, rapid testing for syphilis at the first consultation, at the beginning of the third trimester, and at the time of delivery^6^ ^,^ ^8^ ^-^ ^12^.

Timely and appropriate treatment with benzathine penicillin for pregnant women and their sexual partners is also essential, as are post-treatment follow-up, active search for defaulters, recording of test results and treatments in the prenatal card, and notification of cases, among other measures^6^ ^,^ ^8^ ^-^ ^11^.

Given the complexity of these interventions, the importance of Health Care Networks (RAS, acronym in Portuguese) becomes even more evident. These networks are conceived as a polyarchic organization of services that aims to promote the systemic integration of actions through the provision of continuous, comprehensive, and quality care^10^ ^,^ ^13^.

In this sense, networking is essential to enable the implementation of strategies that favor a comprehensive approach, interprofessional collaborative practice, and the optimization of care flows, especially in the context of prenatal care in Primary Health Care (PHC)^10^ ^-^ ^12^, which acts as a coordinating center for care and a link between the different points of the networks^10^ ^,^ ^13^.

However, although PHC is recognized as the organizer of care, it still faces challenges in coordinating intersectoral and integrated actions that are essential for the prevention of CS^11^ ^-^ ^13^, such as the fragmentation of care in RAS, inequalities in access to health, late diagnosis and treatment, persistent stigma related to sexually transmitted infections (STIs), and inadequate professional training^11^. In addition, many coping strategies are not tailored to local realities, which compromises adherence to and the effectiveness of the plans and policies implemented^11^ ^,^ ^14^.

Despite these challenges, some experiences have proven successful in addressing CS by incorporating educational interventions, government initiatives, and improvement programs^15^ ^-^ ^17^. In this context, nursing, as the largest workforce in the health sector and with a significant presence in PHC, occupies a strategic position. The daily work of nurses in prenatal care allows them to lead interventions aimed at combating CS^18^ ^-^ ^20^, especially by conducting rapid tests, providing prenatal care, and following up on cases^18^ ^-^ ^19^. However, few studies explore the role of nursing in intersectoral planning and in the collective production of strategies to address CS in health networks.

Given the lack of studies that integrate collective construction and strengthen networking in combating CS, this article aims to contribute to the development of care and management practices in health services through a participatory process among RAS professionals and by presenting theoretical, scientific, and practical content that fosters networking. The aim is to ensure the right to humanized care during prenatal care, childbirth, and the postpartum period, as well as the right to child health. From this perspective, we seek to answer the following question: how can we develop strategies to address CS in the RAS? Therefore, the objective was to describe the development of a strategic action plan to address CS, carried out in a participative manner with RAS professionals.

## Method 

### Type of study

A qualitative study^21^ was developed, based on Freire’s theoretical framework^22^, linked to the action research methodology^23^, in accordance with the recommendations of the Consolidated Criteria for Reporting Qualitative Research (COREQ)^24^.

Freire’s theoretical framework, through the problematization of reality and dialogue between historical and social subjects, supports the collective construction of knowledge. This perspective is based on liberatory pedagogy, focused on social transformation, where the active participation of professionals in identifying problems, critically analyzing practices, and developing local solutions dialogues with the concept of education as a practice of freedom^22^.

This framework, integrated with the action research methodology, enables the construction of theories and paths originating in the field of study itself, which are subsequently confronted and validated, with the aim of promoting significant changes in the face of a specific problem^23^.

In this way, a strategic action is implemented that requires the active participation of the actors involved in the reality in question, recognized as collaborators for contributing jointly to the construction of the entire process, not only enriching the final results, but promoting engagement, belonging, and sustainable solutions^22^ ^-^ ^23^.

Action research, characterized as a continuous process of action-reflection-action, is operationalized through 12 phases that occur simultaneously, flexibly, and non-linearly^23^, as shown in Figure 1.

### Study location

The study was conducted in a medium-sized municipality in the Midwest of the state of São Paulo, Brazil, with an estimated population of 74,779 inhabitants^25^, due to its representativeness within a health micro-region comprising eight municipalities. The hierarchical and regionalized structure of health services, with different levels of complexity, makes it a strategic location for analyzing the functioning of the RAS and understanding how healthcare policies and practices are organized in authentic contexts, especially in medium-sized municipalities.

The municipality studied has 73.7% PHC coverage, with an estimated 56.1% population coverage^26^, including five Basic Health Units in the traditional model with a Community Health Agent Programs and eight in the Family Health Strategy model. It also has a multidisciplinary team (eMulti) and specialized care services, such as STI and high-risk prenatal clinics and a maternity ward. PHC and medium-complexity services are managed by a Social Health Organization, while high-complexity services are managed by a philanthropic institution.

The municipality under study presents challenges that led to the development of the research, such as underreporting of cases, inadequate or unprovided treatment, late diagnosis, fewer than six prenatal consultations, and unfavorable outcomes such as abortion, stillbirth, and infant mortality, which highlighted the fragility of care for pregnant women and newborns.

### Data collection period

Data collection was conducted between April and August 2023. 

### Participants

The research collaborators included all health professionals with higher education degrees working in the RAS in this municipality, totaling 80 collaborators, including: social workers, nurses, and doctors working in PHC services of medium and high complexity; nutritionists, psychologists, and physical education professionals from eMulti; members of the Maternal, Infant, and Fetal Mortality Committee; managers from the Municipal Health Secretariat working in the departments of Epidemiological Surveillance, Public Health, Evaluation, and Control. In addition, given the representativeness in the RAS, a counselor/nurse from the Regional Nursing Council of São Paulo and a nurse who acts as a liaison between PHC and the Regional Health Directorate.

### Selection criteria and definition of participants

The selection criterion for collaborators was convenient. The invitation was extended to the 80 professionals eligible for the study, considering as inclusion criteria being a health professional with a higher education degree, providing direct and/or indirect care to people with GS and CS. As an exclusion criterion during the data collection period, individuals on medical leave or vacation were considered.

To ensure broad participation by professionals in the study, an invitation was sent via WhatsApp with information about the research’s purpose and justification, along with a link to pre-register on Google Forms, offering options for participation in the morning or afternoon shifts to avoid compromising care at the RAS. At the same time, the Municipal Health Secretariat issued a circular document reinforcing the importance of participation. After identifying those interested in the form, telephone contact was made with those who had not registered to raise awareness. At the end of the pre-registration period, 56 professionals confirmed their interest. A WhatsApp broadcast list was then created to confirm participation, pass on dates and times, and send a new link to validate registration, linked to the Technical Section responsible for issuing certificates.

### Data collection instruments

The instruments used to record information included: a sociodemographic questionnaire to characterize employees, a word wall created through group dynamics, a comment wall from the employees’ point of view, evaluations applied at the end of each meeting, and a field diary. All these forms of data collection were recorded in writing by the employees. It should be noted that the field diary for recording the collective context during the workshops was observed by a psychologist trained by the three researchers who collected the other data. The instruments had not been tested previously.

The assessments and sociodemographic questionnaire were made possible by Google Forms, available via QR code and printed when employees were unable to access them online. For the word wall and assessments, the Wordle application was used, which generates word clouds that visually highlight the words that appear most often in the text and succinctly express the group’s collective idea.

To construct the action plan, a spreadsheet was used as a planning tool to identify the action, potentialities, weaknesses, and proposed intervention, which later culminated in the final instrument with the following information: objectives, actions, schedule, service, and the professional category responsible. 

### Data collection

Data collection followed the 12 phases of action research (Figure 1), with a workshop focused on the relationship between syphilis and practical reality, enabling the development of a strategic intervention targeted at the needs identified by employees in their work.

**Figure 1:** Details of the phases of the action research on the development of strategies to combat congenital syphilis

Phases of action research	Theorization	Development
**I** **Exploratory phase**	Survey of data to be researched, listing emerging problems and actions to be taken.	Integrative review and situational diagnosis based on epidemiological data on syphilis in pregnant women and congenital syphilis.
**II** **Research topic**	Based on the presentation of the practical problem to be addressed/studied.	Meetings with professionals to discuss the issue and reach consensus on strategy development.
**III** **Identification of problems**	Definition of the main problems identified in the exploratory phase.	The meetings allowed the problem to be exposed and raised awareness for decision-making.
**IV** **The place of theory**	Theoretical/scientific basis, consisting of the articulation of the problem with a theoretical framework adapted to the research.	Based on Freire’s theoretical framework, a dialogical and emancipatory process was developed, in which the subjects critically reflected on their reality.
**V** **Hypotheses**	The researcher’s assumption permeates the maintenance of focus on the theme, as the researcher seeks solutions to address the problem.	Employees were encouraged to reflect, enabling the identification of actions, strengths, and weaknesses found in the services.
**VI** **Seminars**	The moment when information is collected, analyzed, and interpreted, leads to guidelines for action that will be tested in practice.	Face-to-face meetings between researchers and collaborators, called workshops, permeate the phases of action research.
**VII** **Fields of observation, sampling**	Delimitation of the field of observation according to its relevance to a given subject.	Employees were intentionally selected based on their involvement with the topic in their areas of expertise.
**VIII** **Data collection**	Characterized by the production of material for analysis and research guidance.	Collection and processing of epidemiological data and content produced in the workshop.
**IX** **Learning**	Associated with the research process, theoretical knowledge, knowledge production, and proposal development.	Simultaneous learning between researchers and collaborators throughout the research period.
**X** **Formal and informal knowledge**	Seeking knowledge through communication structures between the dimensions of the various phases of research.	The construction of formal and informal knowledge occurred in all phases of the workshop’s development.
**XI** **Action plan**	Members engage in discussions that enable them to outline an action that solves a problem.	Development of an action plan to combat congenital syphilis, including objectives, implementation, sectors, and professionals involved.
**XII** **External disclosure**	The results obtained in the research should be accessible and understandable to everyone.	The action plan was subsequently disclosed to the municipal administration and the entire Health Care Network.

The process began with an integrative literature review, which revealed a knowledge gap regarding prenatal strategies and interventions to address CS. This movement corresponded to phases I - Exploratory phase, IV - The place of theory, IX - Learning, and X - Formal and informal knowledge. At the same time, epidemiological data were collected from notifications of GS and CS, allowing for a situational diagnosis of the topic to be made, in accordance with phases I - Exploratory phase, III - Problem identification, and VIII - Data collection.

The workshop was structured through planning with didactic and pedagogical support, including schedule, duration, collaborators, proposal, objectives, development, resources used, and expected outcomes. It was carried out through three weekly in-person meetings, each lasting an average of three and a half hours. The activity was offered to two classes in the morning and afternoon, encouraging greater employee participation without compromising the continuity of care services. This stage corresponded to phases III - Problem identification, IV - The place of theory, V - Hypotheses, VI - Seminars, VIII - Data collection, IX - Learning, X - Formal and informal knowledge, and XI - Action plan.

In summary, the first workshop meeting sought to present the work proposal, build the care flow for regular pregnant women and pregnant women with syphilis in the RAS, and address the issue with a situational diagnosis based on epidemiological data. The second meeting covered the theory, with a scientific basis, and immersion in technical documents and guidance on government protocols, cases triggering GS, and the evolution to CS, with identification of the action, potentialities, and weaknesses observed in the case. Finally, in the third meeting, impressions generated from the case discussions were presented, along with the importance of mandatory reporting and consolidation of planning, including objectives, actions, scheduling, professional category, and responsible service, which enabled the construction of the final product, which enabled both classes (morning and afternoon) to construct the final product.

### Data analysis

The quantitative data obtained from the sociodemographic questionnaire used to characterize the employees were analyzed using statistical techniques. The information obtained from the word wall, created through collective dynamics, the comment wall, the evaluations carried out at the end of each meeting, and the field diary were organized and interpreted using the content analysis technique, as proposed by Bardin, adapted for research guided by the action research methodology^27^ ^-^ ^28^.

The content analysis began with a cursory reading of all the material generated in the workshop, followed by the formulation of provisional hypotheses and the transformation of most of the text into units of record, which were associated with themes/units of meaning. Thus, each theme/unit of meaning was composed of a set of units of record. Based on the determination and quantification of the themes/units of meaning, the dimensions of emergence were defined, grouped according to the analysis hypotheses, completing the construction of categories^27^ ^-^ ^28^.

To ensure employee confidentiality, an alphanumeric code consisting of the letter “C” followed by a number was assigned to excerpts from the workshop’s content analysis. The symbol [...] was used when a part was omitted.

The data were analyzed within Freire’s theoretical framework^22^, which values the active participation of individuals in the construction of knowledge, encouraging them to reflect on their reality and transform it through collective and committed action.

### Ethical considerations

Participants received an Informed Consent Form outlining the research objectives and voluntary participation. Anonymity, confidentiality, and freedom to withdraw at any time without prejudice were guaranteed. Activities were conducted in welcoming environments, with active listening and respect, aiming to avoid any discomfort or embarrassment. Approval was obtained from the Research Ethics Committee under Opinion No. 5,977,502/CAAE 66686923.7.0000.5411 on March 31, 2023.

## Results

Fifty-six health professionals (70,0% of those invited) participated in this study, mostly women (46) and cisgender individuals (55), with an average age of 37.5 years. Most were married or in a stable relationship (33).

In terms of training, 33 nurses, 12 doctors, eight social workers, one psychologist, one nutritionist, and one physical education professional participated. Of these, 22 had specializations, three had master’s degrees, and one had a doctorate; 16 held management positions. Their length of service ranged from 6 to 30 years (average of 9). Regarding syphilis, 33 had participated in training courses, 29 were qualified to perform rapid tests, and 36 considered themselves capable of managing syphilis in pregnant women (GS) and 34 in CS.

The analysis of the data generated during the workshop meetings resulted in three categories: Getting to know the group and raising awareness among the group; Theoretical basis and applicability in practice; Building strategies to tackle congenital syphilis (Figure 2).

**Figure 2:** Summary of categories that emerged regarding the development of strategies to combat congenital syphilis

Themes/ Units of Meaning	Unit Registration/ Theme n (%)	Category	Unit Registration/ Category n (%)
Relevance of the topic, opportunities for learning and exchanging experiences, sense of belonging, and satisfaction in participating in this workshop.	226 (29.2)	“Getting to know and raising awareness among the group”	286 (37,0)
The presence of healthcare professionals who make up the Healthcare Network, the importance of teamwork, and the value of participatory meetings.	60 (7.8)		
Lack of knowledge about syphilis and uncertainty regarding care for syphilis in pregnant women and congenital syphilis, identifying weaknesses and discrepancies in compliance with protocols and procedures.	68 (8.8)	“Theoretical basis and applicability in reality”	136 (17.6)
Understanding the Healthcare Network and reflecting on the complexity of caring for pregnant women with syphilis.	32 (4.1)		
Knowing the epidemiological data on cases of syphilis in pregnant women and congenital syphilis, periodic dissemination to the Health Care Network.	36 (4.7)		
Implementing strategies to combat syphilis in pregnant women and congenital syphilis, with the aim of changing working practices.	98 (12.7)	“Developing strategies to combat congenital syphilis”	350 (45.4)
Hold more meetings at the workshop, develop other events covering the topic, extend to other professional categories in the Healthcare Network, and create a permanent working group on Syphilis in Pregnant Women and Congenital Syphilis.	77 (10.0)		
Satisfaction in collaborating on the development of a plan, confidence with the emergence of new perspectives for change, and reflections on the care of pregnant women with syphilis.	175 (22.7)		
Total	772 (100.0)		

The category “Getting to know and raising awareness among the group” covered the process of welcoming, listening, and collective construction, revealing the value placed on the space for exchange, the subjective mobilization of professionals, and the feeling of belonging to the proposal. Noteworthy is the active adherence to the work proposal, the recognition of the importance of the theme, and the engagement with the workshop activities: *We are having the opportunity to exchange experiences and ideas to improve the quality of care* [...] (C25); [...] *it’s great that there was space for dialogue and sharing, including experiences that bring difficulties to the work* (C40).

The involvement of professionals from different areas of the RAS was seen as a catalyst for reflection and the proposal of team actions and strategies: *The entire network is participating in the workshop, as each person’s knowledge adds value and provides information necessary for improving care* […] (C27); *Various segments and professionals within the health sector have joined, as this enriches the workshop, bringing more experiences and expertise to everyone* [...] (C40).

The category “Theoretical basis and applicability in reality” comprised the theoretical articulation with the reality experienced by workshop participants, who reported gaps in knowledge about syphilis, uncertainty in management, and divergences in the practices adopted between RAS points, raising awareness of the need for change: *That “the” health network has divergences and differences in the care of pregnant women with syphilis* (C25); *We do not have all the resources or protocols being followed in our municipality* (C23).

The analysis of practices and the functioning of the network made it possible to recognize the complexity of caring for pregnant women with syphilis and the importance of well-defined care flows: *There is a protocol and a flow, and it must be followed so that there are no inconsistencies and risks* [...] (C49)*; That there was a consensus among doctors and nurses regarding prescription autonomy* [...] (C19)*.*

The sharing of local epidemiological data contributed to a critical awareness of the reality of the territory, highlighting the relevance of periodically disseminating this data to RAS services: *The statistical information “served” as a basis for reflecting on our actions* (C25); [...] *it is a pity that this rate is still very high, as it is a totally preventable disease* (C21)*.*

The category “Building strategies to combat congenital syphilis” covered the effectiveness of a participatory strategy- building based on collective reflections, enabling the production of a powerful management tool capable of transforming practical reality.

The collaborators expressed their desire to transform reflections and learning into practical actions: *We could convert this learning into a standardized routine of care* (C15); *We could produce a municipal protocol for prevention and treatment* (C23); *If a specific protocol or follow-up is created, everyone will work in the same way* (C26).

A proposal also emerged to hold periodic meetings to address the issue with the participation of other professionals working in the RAS, in addition to creating a permanent working group on GS and CS: *Create a permanent working group* (C2)*;* [...] *hold periodic meetings to analyze data in the municipality and adjust strategies when necessary* (C24)*.*

Satisfaction with the construction process, the feeling of shared responsibility, and the beginning of changes in the care of pregnant women with syphilis were evident: *The network is getting stronger, improving communication between sectors and within the team itself, with everyone “speaking the same language”* [...] (C27); *It will be great to achieve the workshop’s goal and, in a few years, reap the rewards of these meetings with a decrease in cases of congenital syphilis* (C48); [...] *Showing reality makes us think, reflect, and see situations that we would not have come into contact with if it weren’t for these meetings* (C55).

The workshop provided employees with knowledge, awareness, and theoretical foundations, and developed a strategic action plan to combat CS, a technical/scientific product detailed in 19 objectives: six aimed at all pregnant women and 13 at pregnant women with syphilis. Each objective is detailed in a set of actions, with scheduling, description of services, and professional categories involved, constituting an instrument for practical application.

The objectives of the action plan for all pregnant women were: early identification of pregnancy for timely initiation of prenatal care (before 12 weeks); nursing consultations during prenatal care at all health units; rapid testing for HIV, syphilis, and hepatitis B and C at the first prenatal consultation; repeat rapid testing in 30 days in cases of exposure in the last three months; perform rapid testing in the second and third trimesters and at the time of delivery; and perform prenatal care for the partner.

The objectives for pregnant women with syphilis included: diagnosing, prescribing, and treating syphilis in a timely manner according to the clinical stage; requesting a non-treponemal Venereal Disease Research Laboratory (VDRL) test with sample collection at the time of diagnosis; reporting concurrently with diagnosis; identifying cases of serological scarring from syphilis; providing care to the pregnant woman’s partner; notifying partners if syphilis is confirmed; recording and monitoring the administration of penicillin to pregnant women and partners; collecting and monitoring monthly VDRL tests to check for cure or reinfection; actively searching for missing pregnant women and partners; keeping the treatment history and monitoring of VDRL tests for pregnant women with syphilis up to date with the relevant services; define laboratory workflow regarding the proper completion of test requests and compliance with the syphilis diagnosis flowchart (reverse approach); correctly interpret the syphilis diagnosis flowchart regarding the use of confirmatory treponemal testing; and ensure the proper management of pregnant women with syphilis and newborns at the time of delivery.

The timing of the actions was planned based on short-, medium-, and long-term parameters. Considering the start of implementation, short-term actions refer to the most urgent demands, which are easy to implement and are expected to occur within the first three months of implementation; medium-term actions cover activities that require more time for organization and development, with execution expected within six months; long-term actions require more resources, continuity, and time, and are scheduled to occur within 12 months.

The services involved in developing the actions were represented by the primary healthcare areas and management of the departments of Public Health Strategy, Epidemiological Surveillance, and the Municipal STI/AIDS/Viral Hepatitis Program. The professional categories involved: community health workers, nurses, doctors, eMulti professionals, and RAS service managers. 

## Discussion

The proposal for a participatory construction of strategies to address CS, grounded in action research and Freire’s theoretical framework, enabled an educational and transformative process among researchers and collaborators. This process involved identifying challenges, problematizing reality, and collectively building solutions through the articulation between theory and practice, which enabled the design of an action plan to achieve changes in the perspective of the work shared by RAS in addressing CS.

The workshop provided a space for horizontal dialogue that brought together technical and scientific knowledge and knowledge derived from work practice, encouraging the exchange of experiences across different professional categories and points in the network to promote mutual learning and collective reflection. When different professional categories express their points of view in dialogue, combined with scientific literature and the experience of the subjects, the development of critical concepts and ideas^29^ ^-^ ^30^ is mobilized, capable of transforming reality through praxis.

The problematization of local reality based on epidemiological data, combined with the collaborators’ concrete experience, enabled the activation of critical awareness. This movement of awareness enabled the recognition of the network’s weaknesses, such as the lack of coordination between services, low adherence to protocols, and lack of knowledge of technical documents, as well as local potentialities, such as intersectoral work and the commitment of professionals.

The discussion also revealed a tension between the perception of professional competence and epidemiological and regulatory data. Some collaborators reported feeling prepared to manage CS, while local indicators and reports of inappropriate conduct suggest otherwise. This contradiction may reflect gaps in training, outdated knowledge, or even the normalization of practices that do not adhere to evidence-based standards, in which the perception of sufficiency does not guarantee adherence to evidence-based practice, making it necessary to encourage critical reflection on one’s own practice^20^ ^,^ ^31^.

Proper management of syphilis infection during pregnancy is essential to reduce maternal and neonatal complications, as well as to prevent vertical transmission to the fetus. Thus, health professionals must be up to date on the diagnosis, treatment, and follow-up of GS according to national and international guidelines^6^ ^,^ ^15^ ^,^ ^19^ ^,^ ^31^. However, a considerable number of employees stated that they did not feel capable of managing GS and CS, nor were they familiar with technical documents guiding the care/management of this condition and care flows between different points of the RAS.

The difficulty in accessing technical documents and protocols can represent a critical barrier in the organization of teamwork. Outdated protocols that are not shared, written in inaccessible language, or not incorporated into routine services are significant obstacles. Studies highlight that incorporating educational technologies into practice promotes team adherence and updating^16^ ^-^ ^17^ ^,^ ^19^ ^,^ ^31^, in addition to the adoption of simple digital technologies, such as QR codes, computerized systems integrated with care flows, adapted visual materials, and periodic workshops based on updated protocols.

Inconsistency in the application of clinical protocols highlights recurring problems in health services. When guidelines are poorly disseminated or understood, failures in diagnosis and treatment can occur, with a consequent negative impact on health care^17^ ^,^ ^31^ ^-^ ^32^. In addition, the fragility in compliance with protocols and divergences in conduct highlight the need for standardization and reinforcement in the dissemination of clinical guidelines and protocols, which promotes the improvement and performance of professionals, as well as strengthens the population’s confidence in the health system, favoring better clinical outcomes and safer and more qualified care^17^ ^,^ ^31^ ^-^ ^32^.

To overcome this weakness, it is recommended to invest in continuing and lifelong education using active methodologies^33^ ^-^ ^34^, disseminate simplified and accessible educational materials in print and digital formats^35^ ^-^ ^36^, and strengthen networking by promoting discussions based on real cases^37^ ^-^ ^39^. The systematization of conduct enables all professionals to follow evidence-based recommendations, minimizing variations in care and ensuring safety in the assistance provided.

In this context, participatory workshops expressed in the continuous cycle of action-reflection-action are initiatives that reinforce dialogue and provide a solid basis for the acquisition of technical and scientific knowledge to standardize practices and improve the quality of care^33^ ^-^ ^34^ ^,^ ^40^ through collaborative discussions, problem solving, and greater engagement in health work^20^ ^,^ ^37^ ^,^ ^39^ ^,^ ^41^.

Thus, assertive decision-making in response to a realistic proposal integrates teamwork and networking, strengthens communication, and aligns actions across different services^37^ ^-^ ^39^ ^,^ ^41^, thereby improving the quality of the proposed actions and facilitating implementation and monitoring.

The action plan is in the process of concrete implementation, accompanied by systematic monitoring and continuous evaluations. The effectiveness of this process is already beginning to show in service routines, using quantitative and qualitative indicators that measure adherence, effectiveness, and the impact of the actions developed. Among the main indicators adopted, the following stand out: timely testing (1st, 3rd trimester, and delivery), time between diagnosis and start of treatment, proportion of pregnant women treated with the appropriate benzathine penicillin regimen, treatment of sexual partners, number of notifications, fetal/neonatal deaths attributable to congenital syphilis, and incidence rate per thousand live births^6^ ^,^ ^42^ ^-^ ^43^.

The practice also provides local spaces for discussing results and for continuous review of actions, ensuring the plan’s sustainability and adaptation to the realities of the network. The clear definition of deadlines and professional responsibilities involved has enabled greater organization and effectiveness of the proposal. This alignment between planning and execution, anchored in the experience of professionals at different points in the RAS, ensures a positive and lasting impact, with more efficient resource management and greater potential for transformation in tackling congenital syphilis^20^.

The study has limitations related to convenience sampling, which may have excluded less engaged professionals, and to its single-municipality setting, which limits the generalizability of the results. The workshop took place in three meetings, as planned, but a larger number could have deepened the proposals and included health service users. It is suggested that future studies adopt broader participatory methodologies, with a greater number of meetings and direct involvement of the users.

The contributions lie in the possibility of developing an educational process that combines theory and practice through participatory workshops. In addition, it demonstrates the potential of praxis as a tool for transformation, promoting the valorization of local knowledge, strengthening networking, and identifying the weaknesses and strengths of RAS. Furthermore, it identified concrete ways to overcome barriers, including gaps in service coordination, training gaps, and low adherence to clinical protocols. Finally, the systematization of the action plan, with the definition of goals, indicators, and the actors involved, provides a solid basis for continuous monitoring and evaluation, enabling its replication in other local contexts, provided that the unique characteristics of each territory are respected.

## Conclusion

The participatory development of a strategic action plan to tackle CS with RAS professionals emerged as a concrete result of an educational and transformative process, oriented towards critical and reflective practice, which promoted the autonomy and co-responsibility of professionals. The integration of diverse perspectives and knowledge, derived from practical experience and technical-scientific expertise, enabled the formulation of a feasible, achievable, and affordable network response that covers everything from care to service management.

The meetings highlighted the value of collaborative learning and horizontal dialogue, while revealing gaps in training and in keeping up to date with CS management. This finding reinforces the importance of cooperation and networking, as well as the need for health education actions anchored in teams’ daily routine and linked to clinical guidelines and the health needs of users, families, and communities. Thus, participatory and integrated strategies, such as those developed here, have the potential to strengthen the response to CS and contribute effectively to the improvement of comprehensive care in the SUS.

## Data Availability

All data generated or analysed during this study are included in this published article.
