# Supplementary material for: Participatory development of strategies to combat congenital syphilis: action research
Source: Rev Lat Am Enfermagem. 2026 Aug 10;34:e4894. doi: 10.1590/1518-8345.8014.4894 (PMC13459726; doi:10.1590/1518-8345.8014.4894)
Supplement: Translation

# Translation: Construção participativa de estratégias para o enfrentamento da sífilis congênita: pesquisa-ação^*^

**DOI:** 10.1590/1518-8345.8014.4895

**Authors:** Juliana Sanches Ravagnani, Juliane Andrade, Lucas Cardoso dos Santos, Rúbia Aguiar Alencar

**Affiliations:** 1 Universidade Estadual Paulista, Faculdade de Medicina de Botucatu, Botucatu, SP, Brasil.; 2 Prefeitura de Lins, Secretaria Municipal de Saúde, Lins, SP, Brasil.; 3 Universidade de São Paulo, Escola de Enfermagem, São Paulo, SP, Brasil.

**Keywords:** Sífilis Congênita, Rede de Cuidados Saúde, Pesquisa-Ação, Planejamento em Saúde, Conjunto de Intervenções, Atenção Primária à Saúde

## Abstract

**Objetivo:**

descrever a construção de um plano de ações estratégicas para o enfrentamento da sífilis congênita, realizado de forma participativa com profissionais da Rede de Atenção à Saúde.

**Método:**

pesquisa qualitativa, com abordagem da pesquisa-ação, fundamentada no referencial freireano. Realizada em município do interior paulista, envolveu 56 profissionais de saúde. A ausência de estratégias locais e dados epidemiológicos orientou a realização de uma oficina em três encontros. As atividades foram registradas por murais, avaliações escritas e diário de campo, analisadas por meio da Análise de Conteúdo.

**Resultados:**

os encontros promoveram envolvimento dos profissionais, revelaram fragilidades no manejo da sífilis e impulsionaram a formulação de propostas, como reestruturação dos fluxos de testagem e diagnóstico, prescrição e tratamento oportuno, busca ativa, exames de monitoramento, tratamento de parcerias e manejo adequado dos casos de sífilis em gestante. Emergiram três categorias e, como produto final, foi elaborado o plano de ações estratégicas para o enfrentamento da sífilis congênita com aplicabilidade prática nos serviços da rede.

**Conclusão:**

o processo colaborativo fortaleceu a autonomia e corresponsabilização dos profissionais, integrando saberes técnicos e experiências locais. O plano tem sido implementado e contribuído na qualificação da resposta à sífilis congênita no âmbito do Sistema Único de Saúde.

## Destaques

**(1)** Plano de ações estratégicas para o enfrentamento da sífilis congênita. **(2)** Pesquisa-ação como abordagem metodológica que permite a construção participativa. **(3)** Estratégias participativas e integradas fortalecem o enfrentamento da sífilis. **(4)** Importância da cooperação e da sistematização do trabalho em rede. **(5)** Resposta em rede à sífilis congênita, factível, exequível e de custo acessível.

## Introdução 

A sífilis é uma infecção bacteriana sexualmente transmissível, evitável e curável, que tem se apresentado como uma preocupação crescente em termos de saúde pública global. A Organização Mundial da Saúde (OMS) estima que o número de casos aumentou significativamente nos últimos anos, com variações expressivas entre as diferentes regiões do mundo^1^ ^-^ ^3^. 

As Américas, com disparidades entre América do Norte (maior incidência), América Latina e Caribe, concentram a maior carga global da doença, com cerca de 3,37 milhões de novos casos, o que corresponde a 42,0% das infecções recentes, seguidas por regiões da África e do Sudeste Asiático^1^ ^,^ ^3^ ^-^ ^4^. Essa tendência reflete diretamente no aumento dos casos de sífilis congênita (SC), com aproximadamente 68 mil nascidos vivos afetados por ano no mundo^1^ ^,^ ^3^ ^,^ ^5^, com amplo espectro de manifestações clínicas e desfechos adversos como aborto espontâneo, morte fetal, prematuridade, baixo peso ao nascer, anomalias congênitas e sequelas neurológicas^5^ ^-^ ^8^.

Nesse contexto, o enfrentamento da SC exige condições favoráveis para o desenvolvimento de ações e estratégias voltadas à qualificação da atenção à saúde no pré-natal e puerpério. Entre essas, destacam-se a implementação de políticas públicas de saúde, o comprometimento de gestores, o trabalho em equipe e em rede, além do cuidado pré-natal adequado com captação precoce e vinculação com o serviço, testagem rápida para sífilis na primeira consulta, no início do terceiro trimestre e no momento do parto^6^ ^,^ ^8^ ^-^ ^12^. 

Também são fundamentais o tratamento oportuno e adequado com penicilina benzatina para as gestantes e suas parcerias sexuais, assim como seguimento pós-tratamento, busca ativa de faltosos, registro dos resultados de exames e tratamentos no cartão de pré-natal e a notificação dos casos, dentre outras^6^ ^,^ ^8^ ^-^ ^11^. 

Considerando a complexidade dessas intervenções, ganha relevo a importância das Redes de Atenção à Saúde (RAS), concebidas como uma organização poliárquica de serviços que visa promover a integração sistêmica de ações por meio da oferta de atenção contínua, integral e de qualidade^10^ ^,^ ^13^. 

Nesse sentido, o trabalho em rede é fundamental para viabilizar a implementação de estratégias que favoreçam uma abordagem integral, uma prática colaborativa interprofissional e a otimização de fluxos assistenciais, especialmente no contexto do pré-natal na Atenção Primária à Saúde (APS)^10^ ^-^ ^12^, que atua como centro coordenador do cuidado e articulador entre os diferentes pontos das redes^10^ ^,^ ^13^.

Entretanto, embora a APS seja reconhecida como ordenadora do cuidado, ainda enfrenta desafios para coordenar ações intersetoriais e integradas, essenciais à prevenção da SC^11^ ^-^ ^13^, como a fragmentação do cuidado nas RAS, as desigualdades no acesso à saúde, o diagnóstico e tratamento tardios, o persistente estigma relacionado às Infecções Sexualmente Transmissíveis (IST) e a frágil formação profissional^11^. Ademais, observa-se que muitas estratégias de enfrentamento não se articulam às realidades locais, o que compromete a adesão e a efetividade dos planos e políticas implementados^11^ ^,^ ^14^. 

Apesar desses desafios, algumas experiências têm se mostrado exitosas no enfrentamento da SC ao incorporarem intervenções educacionais, iniciativas governamentais e programas de melhorias^15^ ^-^ ^17^. Nesse contexto, a enfermagem, por ser a maior força de trabalho do setor saúde e ter presença significativa na APS, ocupa posição estratégica. A atuação cotidiana de enfermeiros no pré-natal permite que liderem intervenções voltadas ao enfrentamento da SC^18^ ^-^ ^20^, especialmente na realização de testes rápidos, acompanhamento pré-natal e seguimento de caso^18^ ^-^ ^19^. No entanto, são escassos os estudos que exploram o papel da enfermagem no planejamento intersetorial e na produção coletiva de estratégias para o enfrentamento da SC em redes de saúde.

Em face da lacuna de estudos que integram a construção coletiva e o fortalecimento do trabalho em rede no enfrentamento da SC, este artigo propõe-se a contribuir com a construção de práticas assistenciais e de gestão nos serviços de saúde, a partir de um processo participativo entre profissionais da RAS e da apresentação de conteúdo teórico, científico e prático que favoreçam o trabalho em rede. Busca-se, assim, assegurar o direito à atenção humanizada no pré-natal, parto e puerpério, bem como à saúde da criança. A partir dessa perspectiva, pretende-se responder à seguinte pergunta: como construir estratégias para o enfrentamento da SC na RAS? Logo, o objetivo foi descrever a construção de um plano de ações estratégicas para o enfrentamento da SC, realizado de forma participativa com profissionais da RAS.

## Método 

### Tipo de estudo

Desenvolveu-se estudo de natureza qualitativa^21^, fundamentado no referencial teórico freireano^22^, articulado à metodologia da pesquisa-ação^23^, atendendo às recomendações do *Consolidated Criteria for Reporting Qualitative Research* (COREQ)^24^.

O referencial teórico freireano, por meio da problematização da realidade e do diálogo entre sujeitos históricos e sociais, sustenta a construção coletiva do conhecimento. Essa perspectiva se apoia na pedagogia libertadora, voltada para a transformação social, onde a participação ativa dos profissionais na identificação de problemas, na análise crítica das práticas e na elaboração de soluções locais dialoga com a concepção de educação como prática da liberdade^22^.

Esse referencial integrado à metodologia da pesquisa-ação possibilita a construção de teorias e caminhos originados no próprio campo de estudo, os quais são posteriormente confrontados e validados, com o objetivo de promover mudanças significativas diante de uma problemática específica^23^.

Dessa maneira, implementa-se uma ação estratégica que exige a participação ativa dos atores envolvidos na realidade em questão, reconhecidos como colaboradores por contribuírem de forma conjunta na construção de todo o processo, não apenas enriquecendo os resultados finais, mas promovendo o engajamento, o pertencimento e soluções sustentáveis^22^ ^-^ ^23^.

A pesquisa-ação, caracterizada como um processo contínuo de ação-reflexão-ação, é operacionalizada por meio de 12 fases que ocorrem de forma simultânea, flexível e não linear^23^, conforme Figura 1.

### Local do estudo

O estudo foi desenvolvido em um município de médio porte do centro-oeste do estado de São Paulo, Brasil, cuja população é estimada em 74.779 habitantes^25^, devido à sua representatividade dentro de uma microrregião de saúde composta por oito municípios. A estrutura hierarquizada e regionalizada dos serviços de saúde, com diferentes níveis de complexidade, torna o local estratégico para analisar o funcionamento da RAS e compreender como as políticas e práticas de assistência se organizam em contextos reais, especialmente em municípios de médio porte. 

O município do estudo apresenta 73,7% de cobertura da APS, com estimativa de 56,1% de cobertura da população^26^, com cinco Unidades Básicas de Saúde no modelo tradicional com Programa de Agentes Comunitários de Saúde e oito no modelo Estratégia de Saúde da Família. Conta ainda com equipe multiprofissional (eMulti) e serviços de atenção especializada, como ambulatórios de IST e de pré-natal de alto risco e uma maternidade. A gestão dos serviços da APS e de média complexidade é feita por uma Organização Social de Saúde e da alta complexidade por uma instituição filantrópica.

O município de estudo apresenta como desafios que levaram ao desenvolvimento da pesquisa, a subnotificação dos casos, tratamentos inadequados ou não realizados, diagnóstico tardio, número de consultas no pré-natal menor que seis, desfechos desfavoráveis como aborto, natimorto e óbito infantil, o que evidenciou a fragilidade do cuidado à gestante e ao RN.

### Período da coleta de dados

A coleta de dados foi conduzida entre os meses de abril e agosto de 2023. 

### Participantes

Os colaboradores da pesquisa compreenderam todos os profissionais da saúde com nível superior inseridos na RAS deste município, totalizando 80 colaboradores, sendo eles: assistentes sociais, enfermeiros e médicos com atuação nos serviços da APS, média e alta complexidade; nutricionista, psicólogo e profissional de educação física da eMulti; membros do Comitê de Mortalidade Materno, Infantil e Fetal; gestores da Secretaria Municipal de Saúde com atuação nos departamentos de Vigilância Epidemiológica, Saúde Coletiva, Avaliação e Controle. Ainda, ante a representatividade na RAS, uma conselheira/enfermeira do Conselho Regional de Enfermagem de São Paulo e uma enfermeira/articuladora entre APS e Diretoria Regional de Saúde. 

### Critérios de seleção e definição dos participantes 

O critério de seleção dos colaboradores foi por conveniência. O convite foi realizado para os 80 profissionais elegíveis para o estudo, considerando como critérios de inclusão ser profissional de saúde com nível superior, realização de cuidado direto e/ou indireto às pessoas com SG e SC. Como critério de exclusão durante o período de coleta de dados, consideraram-se os indivíduos em licença médica ou em período de férias.

Para garantir ampla participação dos profissionais no estudo, foi enviado um convite via WhatsApp com informações sobre o objetivo da pesquisa, justificativa e um link para pré-inscrição no Google *Forms*, oferecendo opções de participação nos turnos da manhã ou tarde para não comprometer a assistência na RAS. Paralelamente, a Secretaria Municipal de Saúde emitiu um documento circular reforçando a importância da participação. Após identificar os interessados pelo formulário, houve contato telefônico com os não inscritos para sensibilização. Ao final da pré-inscrição, 56 profissionais confirmaram interesse. Em seguida, criou-se uma lista de transmissão no WhatsApp para confirmar a participação, repassar datas e horários, e enviar novo link para validação da inscrição, vinculada à Seção Técnica responsável pela emissão dos certificados.

### Instrumentos de coleta de dados

Os instrumentos utilizados para registro das informações foram: questionário sociodemográfico para caracterização dos colaboradores, mural de palavras confeccionado em dinâmica coletiva, mural de comentários sob o ponto de vista dos colaboradores, as avaliações aplicadas ao final de cada encontro e o diário de campo. Todas estas formas de coleta de dados foram registradas de forma escrita pelos colaboradores. Ressalta-se que o diário de campo para registro do contexto coletivo, no momento das oficinas, foi observado por uma psicóloga treinada pelas três pesquisadoras que coletaram os demais dados. Os instrumentos não foram testados anteriormente. 

As avaliações e o questionário sociodemográfico foram viabilizados pelo Google *Forms,* disponibilizados em *QR code* e impressos na impossibilidade de acesso pelo colaborador. Para o mural de palavras e as avaliações, utilizou-se o aplicativo Wordle, capaz de gerar nuvens de palavras, permitindo a visualização em destaque das palavras que aparecem mais vezes no texto, expressando sinteticamente a ideia do grupo em coletividade.

Para a construção do plano de ação, utilizou-se uma planilha, caracterizada como instrumento de planejamento, contendo a identificação da ação, potencialidades, fragilidades e proposta de intervenção que culminou posteriormente no instrumento final com as seguintes informações: objetivos, ações, aprazamento, serviço e categoria profissional responsável.

### Coleta de dados

A coleta de dados seguiu as 12 fases da pesquisa-ação (Figura 1), realizando-se uma oficina de trabalho baseada na relação da problemática de sífilis com a realidade prática, possibilitando a construção de uma intervenção estratégica direcionada às necessidades identificadas na realidade do trabalho pelos colaboradores.

**Figura 1:** Detalhamento das fases da pesquisa-ação sobre a construção de estratégias para o enfrentamento da sífilis congênita

Fases da pesquisa-ação	Teorização	Desenvolvimento
**I** **Fase exploratória**	Levantamento de dados a serem pesquisados, elencando os problemas emergentes e quais ações a serem realizadas.	Revisão integrativa e diagnóstico situacional com base em dados epidemiológicos sobre sífilis em gestantes e congênita.
**II** **Tema da pesquisa**	Baseado na exposição do problema prático a ser abordado/estudado.	Encontros com profissionais expondo a problemática e consenso sobre construção de estratégias.
**III** **Colocação dos problemas**	Definição dos principais problemas identificados na fase exploratória.	Os encontros permitiram a exposição do problema e a sensibilização para a tomada de decisão.
**IV** **O lugar da teoria**	Embasamento teórico/científico, consistindo na articulação da problemática com um referencial teórico adaptado à pesquisa.	A partir do referencial teórico freireano, desenvolveu-se um processo dialógico e emancipatório, no qual os sujeitos refletiram criticamente sobre sua realidade.
**V** **Hipóteses**	A suposição formulada pelo pesquisador permeia a manutenção do foco na temática, buscando soluções necessárias ao enfrentamento do problema.	Os colaboradores foram levados à reflexão, possibilitando o levantamento de ações, potencialidades e fragilidades encontradas nos serviços.
**VI** **Seminários**	Momento em que as informações serão coletadas, analisadas e submetidas a interpretações, levantando diretrizes para ação que serão testadas na prática.	Encontros presenciais entre pesquisadoras e colaboradores, chamados de oficina de trabalho, permeando as fases da pesquisa-ação.
**VII** **Campos de observação, amostragem**	Delimitação do campo de observação em função de sua relevância para um determinado assunto.	Os colaboradores foram intencionalmente selecionados conforme o envolvimento com a temática em suas áreas de atuação.
**VIII** **Coleta de dados**	Caracterizada pela produção de material para análise e direcionamento da pesquisa.	Levantamento e tratamento dos dados epidemiológicos e conteúdo produzido na oficina de trabalho.
**IX** **Aprendizagem**	Associada ao processo de investigação, conhecimentos teóricos, produção de saberes e construção de propostas.	Aprendizagem simultânea entre pesquisadores e colaboradores durante todo o período da pesquisa.
**X** **Saber formal e informal**	Busca dos saberes por meio de estruturas de comunicação entre as dimensões das diversas fases da pesquisa.	A construção do saber formal e informal ocorreu em todas as fases do desenvolvimento da oficina de trabalho.
**XI** **Plano de ação**	Os integrantes se envolvem em discussões que permitem o delineamento de uma ação que solucione um problema.	Construção de um plano de ação para o enfrentamento da sífilis congênita, contendo objetivos, execução, setores e profissionais envolvidos.
**XII** **Divulgação externa**	O retorno dos resultados obtidos na pesquisa deve ser acessível e compreensível para todas as pessoas.	O plano de ação foi divulgado posteriormente à gestão municipal e a toda a Rede de Atenção à Saúde.

O processo foi iniciado com base em uma revisão integrativa da literatura, que evidenciou uma lacuna no conhecimento relacionado às estratégias e intervenções no pré-natal voltadas ao enfrentamento da SC. Esse movimento correspondeu às fases I - Fase exploratória, IV - O lugar da teoria, IX - Aprendizagem e X - Saber formal e informal. Paralelamente, foi realizado o levantamento de dados epidemiológicos a partir das notificações de SG e SC, permitindo traçar um diagnóstico situacional da temática, em conformidade com as fases I - Fase exploratória, III - Colocação dos problemas e VIII - Coleta de dados.

A oficina de trabalho foi estruturada por meio de planejamento com suporte didático e pedagógico, contendo horário, duração, colaboradores, proposta, objetivos, desenvolvimento, recursos utilizados e produtos esperados. Sua operacionalização ocorreu por meio de três encontros presenciais, semanais e com duração média de três horas e meia cada. A atividade foi ofertada a duas turmas distintas, no período matutino e vespertino, o que favoreceu maior adesão dos colaboradores, sem comprometer a continuidade dos serviços assistenciais. Essa etapa correspondeu às fases III - Colocação dos problemas, IV - O lugar da teoria, V - Hipóteses, VI - Seminários, VIII - Coleta de dados, IX - Aprendizagem, X - Saber formal e informal e XI - Plano de ação.

Sumariamente, o primeiro encontro da oficina buscou a apresentação da proposta de trabalho, construção do fluxo de atendimento da gestante habitual e gestante com sífilis na RAS, e colocação da problemática com diagnóstico situacional pelos dados epidemiológicos. O segundo encontro abrangeu a teoria dialogada com embasamento científico e imersão em documentos técnicos e protocolos governamentais norteadores, casos disparadores de SG com evolução para SC com identificação da ação, potencialidades e fragilidades observadas no caso. Por fim, no terceiro encontro foram apresentadas as impressões geradas a partir das discussões dos casos, importância da notificação compulsória e consolidação do planejamento, este contendo objetivos, ações, aprazamento, categoria profissional e serviço responsável, o que possibilitou a construção do produto final pelas duas turmas (matutino e vespertino).

### Análise dos dados

Os dados quantitativos obtidos por meio do questionário sociodemográfico, utilizados para a caracterização dos colaboradores, foram analisados com base em técnicas estatísticas. Já as informações provenientes do mural de palavras, elaborado em dinâmica coletiva, do mural de comentários, das avaliações realizadas ao final de cada encontro e do diário de campo foram organizadas e interpretadas por meio da técnica de análise de conteúdo, conforme proposta por Bardin, adaptada para pesquisas orientadas pela metodologia da pesquisa-ação^27^ ^-^ ^28^.

A análise de conteúdo foi iniciada com a leitura flutuante de todo o material gerado na oficina, seguida de formulação de hipóteses provisórias e transformação da maior parte do texto em unidade de registro, estas associadas em temas/unidades de significação. Assim, cada tema/unidade de significação foi composto por um conjunto de unidades de registro. A partir da determinação e quantificação dos temas/unidades de significação, foram definidas as dimensões de surgimento, agrupando-as segundo as hipóteses de análise, perfazendo a construção de categorias^27^ ^-^ ^28^. 

Para garantir o sigilo dos colaboradores, atribuiu-se um código alfanumérico composto pela letra “C” sucedida de um algarismo para apresentação de excertos da análise de conteúdo oriundos da oficina de trabalho, o símbolo [...] foi considerado quando alguma parte foi omitida.

Os dados foram analisados à luz do referencial teórico freireano^22^, que valoriza a participação ativa dos indivíduos na construção do conhecimento, incentivando-os a refletir sobre sua realidade e a transformá-la por meio da ação coletiva e comprometida.

### Considerações éticas

Os colaboradores receberam o Termo de Consentimento Livre e Esclarecido com orientações sobre os objetivos da pesquisa e a participação voluntária. Garantiram-se anonimato, sigilo e liberdade para desistência a qualquer momento, sem prejuízos. As atividades foram conduzidas em ambientes acolhedores, com escuta ativa e respeito, visando evitar qualquer desconforto ou constrangimento. Aprovação obtida pelo Comitê de Ética em Pesquisa sob Parecer n.º 5.977.502/CAAE 66686923.7.0000.5411 em 31/03/2023.

## Resultados

Participaram desta pesquisa 56 profissionais de saúde (70,0% dos convidados), majoritariamente mulheres (46) e cisgêneros (55), com média de idade de 37,5 anos. A maioria era casada ou em união estável (33).

Quanto à formação, participaram 33 enfermeiras, 12 médicos, oito assistentes sociais, um psicólogo, um nutricionista e um profissional de educação física. Destes, 22 tinham especialização, três mestrado e um doutorado; 16 ocupavam cargos de gestão. O tempo de atuação variou de 6 a 30 anos (média de 9). Sobre sífilis, 33 haviam participado de capacitações, 29 eram habilitados para teste rápido, e 36 se consideravam aptos ao manejo da sífilis em gestante (SG) e 34 da SC.

A análise dos dados gerada nos encontros da oficina de trabalho resultou em três categorias: Conhecendo e sensibilizando o grupo; Fundamentação teórica e aplicabilidade na realidade; Construindo estratégias para o enfrentamento da sífilis congênita (Figura 2).

**Figura 2:** Síntese da emersão de categorias sobre a construção de estratégias para o enfrentamento da sífilis congênita

Temas/ Unidades de Significação	Unidade Registro/ Tema n (%)	Categoria	Unidade Registro/ Categoria n (%)
Relevância da temática, oportunidade de aprendizado e troca de experiências, sensação de pertencimento e satisfação em participar desta oficina.	226 (29,2)	“Conhecendo e sensibilizando o grupo”	286 (37,0)
A presença dos profissionais de saúde que compõem a Rede de Atenção à Saúde, a importância do trabalho em equipe e a valorização de encontros participativos.	60 (7,8)		
Deficiência no conhecimento sobre sífilis e insegurança no cuidado sobre Sífilis em Gestante e Congênita, identificando as fragilidades e divergências no cumprimento de protocolos e condutas.	68 (8,8)	“Fundamentação teórica e aplicabilidade na realidade”	136 (17,6)
Compreendendo a Rede de Atenção à Saúde e refletindo sobre a complexidade do cuidado às gestantes com sífilis.	32 (4,1)		
Conhecendo os dados epidemiológicos sobre os casos de Sífilis em Gestante e Congênita, divulgação periódica para a Rede de Atenção à Saúde.	36 (4,7)		
Efetivando o desenvolvimento de estratégias para o enfrentamento da Sífilis em Gestante e Congênita, com o objetivo de mudança na prática do trabalho.	98 (12,7)	“Construindo estratégias para o enfrentamento da Sífilis Congênita”	350 (45,4)
Dispor de mais encontros na oficina, desenvolver outros eventos que abarquem a temática, extensão para outras categorias profissionais da Rede de Atenção à Saúde, criação de grupo permanente de trabalho sobre Sífilis em Gestante e Congênita.	77 (10,0)		
Satisfação em colaborar com a construção de um plano, segurança com início de novas perspectivas de mudanças e reflexões em relação ao cuidado à gestante com sífilis.	175 (22,7)		
Total	772 (100,0)		

A categoria “Conhecendo e sensibilizando o grupo” contemplou o processo de acolhimento, escuta e construção coletiva, revelando a valorização do espaço de troca, a mobilização subjetiva dos profissionais e o sentimento de pertencimento à proposta. Destaca-se a adesão ativa à proposta de trabalho, o reconhecimento da importância do tema e o engajamento com as atividades da oficina: *Estamos tendo a oportunidade de trocar experiências e ideias para melhorar a qualidade do atendimento* [...] (C25); [...] *que bom que houve espaço para diálogo e compartilhar inclusive experiências que trazem dificuldade ao trabalho* (C40).

O envolvimento de profissionais que compõem os diferentes pontos da RAS foi percebido como um elemento potencializador para a reflexão e a proposição de ações e estratégias em equipe: *A rede completa está participando da oficina, pois o conhecimento de cada um agrega e traz informações necessárias para melhoria do atendimento* […] (C27); *Houve adesão de diversos segmentos e profissionais dentro da saúde, pois isso enriquece a oficina, trazendo mais vivências e experiência para todos* […] (C40).

A categoria “Fundamentação teórica e aplicabilidade na realidade” compreendeu a articulação teórica com a realidade vivida pelos colaboradores da oficina que relataram lacunas no conhecimento sobre sífilis, insegurança no manejo e divergências nas práticas adotadas entre os pontos da RAS, trazendo a sensibilização da necessidade de mudanças: *Que “na” rede de saúde existem divergências e diferenças na assistência de gestante com sífilis* (C25); *Não temos todos os recursos ou protocolos sendo cumprindo no nosso município* (C23).

A análise das práticas e o funcionamento do trabalho em rede permitiram reconhecer a complexidade do cuidado às gestantes com sífilis e a importância de fluxos assistenciais bem definidos: *Existe um protocolo e um fluxo e ele deve ser seguido para que não haja inconsistências e riscos* [...] (C49); *Que se houvesse um consenso entre médicos e enfermeiros quanto a autonomia de prescrição* [...] (C19).

O compartilhamento dos dados epidemiológicos locais contribuiu para uma conscientização crítica da realidade do território, ressaltando a relevância de divulgação periódica destes dados aos serviços da RAS: *As informações estatísticas “serviu” de base para refletir nossas ações* (C25); [...] *é uma pena que esse índice ainda é muito alto, por ser uma doença totalmente evitável* (C21).

A categoria “Construindo estratégias para o enfrentamento da Sífilis Congênita” abrangeu a efetividade na construção participativa de estratégias, a partir das reflexões coletivas, permitindo a produção de uma potente ferramenta gerencial, capaz de transformar a realidade prática. 

Os colaboradores manifestaram o desejo de transformar as reflexões e o aprendizado em ações práticas: *Pudéssemos converter esse aprendizado em uma rotina padronizada de assistência* (C15); *Produzirmos um protocolo municipal, de prevenção e tratamento* (C23); *Se criar um protocolo ou seguimento específico que todos trabalhem da mesma forma* (C26).

Também emergiu a proposta de criação de encontros periódicos que abordassem a temática com a participação de outros profissionais atuantes na RAS, além da criação de grupo permanente de trabalho sobre SG e SC: *Criar um permanente grupo de trabalho* (C2); [...] *realizar encontros periódicos para análise de dados no município e elaboração de ajuste em estratégias quando necessário* (C24).

A satisfação com o processo de construção, o sentimento de corresponsabilidade e o início de mudanças em relação ao cuidado à gestante com sífilis foram evidentes: *A rede se fortalecer, melhorando a comunicação entre setores e entre a própria equipe, todos “falando a mesma língua”* [...] (C27); *Será ótimo atingirmos o objetivo da oficina e daqui alguns anos, colher os frutos desses encontros com a diminuição dos casos de sífilis congênita* (C48); [...] *Mostrar a realidade nos faz pensar, refletir e ver situações que não teríamos contato se não houve esses encontros* (C55).

A oficina de trabalho oportunizou aos colaboradores conhecimento, sensibilização, fundamentação teórica e a construção de um plano de ações estratégicas para o enfrentamento da SC, caracterizado como um produto técnico/científico, detalhado em 19 objetivos, sendo seis voltados a todas as gestantes e 13 às gestantes com sífilis. Cada objetivo está pormenorizado em um conjunto de ações, com aprazamento, descrição dos serviços e categorias profissionais envolvidas, constituindo-se um instrumento para aplicação na prática.

Os objetivos do plano de ação para todas as gestantes foram: identificar precocemente a gestação para início oportuno do pré-natal (antes 12 semanas); realizar consulta de enfermagem no pré-natal em todas as unidades de saúde; realizar a testagem rápida para HIV, sífilis e hepatites B e C na primeira consulta de pré-natal; repetir a testagem rápida em 30 dias em situação de exposição nos últimos três meses; realizar a testagem rápida no segundo e terceiro trimestres e no momento do parto; e, realizar o pré-natal da parceria.

Já os objetivos voltados às gestantes com sífilis abarcaram: diagnosticar, prescrever e tratar oportunamente a sífilis de acordo com a fase clínica; solicitar teste não treponêmico *Venereal Disease Research Laboratory* (VDRL) com coleta da amostra no momento do diagnóstico; notificar concomitantemente ao diagnóstico; identificar os casos de cicatriz sorológica da sífilis; realizar cuidado a parceria da gestante; notificar parceria se confirmada a sífilis; registrar e monitorar a administração da penicilina na gestante e parceria; coletar e monitorar o exame de VDRL mensal para controle de cura ou reinfecção; realizar busca ativa das gestantes faltosas e parcerias; manter atualizado o histórico de tratamento e monitoramento do exame de VDRL da gestante com sífilis junto aos serviços interessados; definir fluxo laboratorial quanto ao adequado preenchimento da solicitação de exame e cumprimento do fluxograma de diagnóstico da sífilis (abordagem reversa); interpretar corretamente o fluxograma de diagnóstico da sífilis, quanto à utilização do exame treponêmico confirmatório; e garantir o manejo adequado da gestante com sífilis e do recém-nascido no momento do parto.

O aprazamento da execução das ações foi planejado com base nos parâmetros de curto, médio e longo prazo. Considerando como início a implantação, as ações de curto prazo referem-se às demandas mais urgentes, de fácil implementação, previstas para ocorrer dentre os três primeiros meses de implementação; as ações de médio prazo abrangem atividades que exigem maior tempo de organização e desenvolvimento, com execução prevista em até seis meses; já as ações de longo prazo demandam mais recursos, continuidade e tempo, sendo programadas para ocorrer até 12 meses.

Os serviços envolvidos no desenvolvimento das ações estiveram representados pelas áreas assistenciais da APS e gestão dos departamentos de Saúde Coletiva, Vigilância Epidemiológica e Programa Municipal de IST/Aids/Hepatites Virais. Já as categorias profissionais envolveram: agentes comunitários de saúde, enfermeiros, médicos, profissionais da eMulti e gestores dos serviços da RAS. 

## Discussão 

A proposta de construção participativa de estratégias para o enfrentamento da SC à luz da pesquisa-ação e fundamentada no referencial teórico freireano viabilizou um processo educativo e transformador entre pesquisadores e colaboradores. Esse processo envolveu a identificação de desafios, a problematização da realidade e a construção coletiva de soluções por meio da articulação entre teoria e prática, o que possibilitou o delineamento de um plano de ação para o alcance de mudanças na perspectiva do trabalho compartilhado pela RAS no tocante ao enfrentamento da SC.

A oficina de trabalho realizada proporcionou um espaço de diálogo horizontal que articulou saberes técnico-científicos e conhecimentos oriundos da prática de trabalho, favorecendo a troca de experiências entre diferentes categorias profissionais e pontos da rede, de forma a promover a aprendizagem mútua e reflexão coletiva. Quando diferentes categorias profissionais expõem seu ponto de vista em exercício de diálogo, combinado com a literatura científica e a experiência dos sujeitos, mobiliza-se a elaboração de concepções e ideias críticas^29^ ^-^ ^30^, capazes de transformar a realidade através da práxis.

A problematização da realidade local a partir dos dados epidemiológicos, combinada à vivência concreta dos colaboradores, permitiu ativar a consciência crítica. Esse movimento de conscientização possibilitou o reconhecimento das fragilidades da rede, como a desarticulação dos serviços, a baixa adesão a protocolos e o desconhecimento de documentos técnicos, bem como das potencialidades locais, como o trabalho intersetorial e o comprometimento dos profissionais.

A discussão também revelou uma tensão entre a percepção de competência dos profissionais e os dados epidemiológicos e normativos. Parte dos colaboradores relatou sentir-se preparada para o manejo da SC, embora os indicadores locais e relatos de condutas inadequadas indiquem o oposto. Essa contradição pode refletir lacunas formativas, desatualização dos saberes ou mesmo naturalização de práticas equivocadas em que a percepção de suficiência não garante adesão a condutas baseadas em evidências, sendo necessário fomentar a reflexão crítica sobre a própria prática^20^ ^,^ ^31^.

O manejo adequado da infecção da sífilis durante a gravidez é essencial para reduzir complicações maternas e neonatais, bem como para evitar transmissão vertical para o feto. Assim, profissionais de saúde devem estar atualizados sobre o diagnóstico, tratamento e acompanhamento da SG conforme diretrizes nacionais e internacionais^6^ ^,^ ^15^ ^,^ ^19^ ^,^ ^31^. Contudo, considerável número de colaboradores declarou não se sentir aptos a realizar o manejo da SG e SC, assim como não conhecer documentos técnicos norteadores do cuidado/manejo do referido agravo e fluxos assistenciais entre os diferentes pontos da RAS.

A dificuldade de acesso aos documentos técnicos e protocolos pode representar uma barreira crítica na organização do trabalho das equipes. Protocolos desatualizados, não compartilhados, com linguagem inacessível ou não incorporados à rotina dos serviços são entraves relevantes. Estudos destacam que a incorporação de tecnologias educacionais à prática favorece a adesão e atualização das equipes^16^ ^-^ ^17^ ^,^ ^19^ ^,^ ^31^, somadas à adoção de tecnologias digitais simples, como *QR codes*, sistemas informatizados integrados com os fluxos assistenciais, materiais visuais adaptados e oficinas periódicas baseadas em protocolos atualizados. 

A inconsistência na aplicação de protocolos clínicos evidencia problemas recorrentes nos serviços de saúde. Quando diretrizes não são bem difundidas ou compreendidas, podem ocorrer falhas no diagnóstico e tratamento, com consequente impacto negativo no cuidado em saúde^17^ ^,^ ^31^ ^-^ ^32^. Além disso, a fragilidade no cumprimento de protocolos e as divergências nas condutas evidenciam a necessidade de padronização e reforço na disseminação das diretrizes clínicas e protocolos, o que promove o aprimoramento e desempenho dos profissionais, como também fortalece a confiança da população no sistema de saúde, favorecendo melhores desfechos clínicos e um atendimento mais seguro e qualificado^17^ ^,^ ^31^ ^-^ ^32^. 

Para superar essa fragilidade, recomenda-se o investimento em ações de educação continuada e permanente a partir de metodologias ativas^33^ ^-^ ^34^, a disseminação de materiais educativos simplificados e acessíveis, em formato impresso e digital^35^ ^-^ ^36^ e o fortalecimento do trabalho em rede, promovendo discussões com base em casos reais^37^ ^-^ ^39^. A sistematização das condutas permite que todos os profissionais sigam recomendações baseadas em evidências, o que minimiza variações no atendimento e garante segurança na assistência prestada. 

Nesse contexto, oficinas participativas expressas no ciclo contínuo de ação-reflexão-ação são iniciativas que reforçam o diálogo e proporcionam uma base sólida para a aquisição de conhecimento técnico-científico para uniformizar as práticas e melhorar a qualidade do atendimento^33^ ^-^ ^34^ ^,^ ^40^ por meio de discussões colaborativas, resolução de problemas e maior engajamento no trabalho em saúde^20^ ^,^ ^37^ ^,^ ^39^ ^,^ ^41^. 

Dessa forma, a tomada de decisão assertiva ante uma proposta realista integra o trabalho em equipe e em rede e fortalece a comunicação, alinhando as ações entre os diferentes serviços^37^ ^-^ ^39^ ^,^ ^41^, colaborando para o aprimoramento da qualidade das ações propostas, bem como facilidade na implementação e monitoramento.

O plano de ação está em processo de implementação concreta, sendo acompanhado por monitoramento sistemático e avaliações contínuas. A efetividade desse processo já começa a se evidenciar na rotina dos serviços, com o uso de indicadores quantitativos e qualitativos que permitem medir a adesão, efetividade e impacto das ações desenvolvidas. Dentre os principais indicadores adotados, destacam-se: testagem em tempo oportuno (1.º, 3.º trimestre e parto), tempo entre diagnóstico e início do tratamento, proporção de gestantes tratadas com o esquema adequado de penicilina benzatina, tratamento da parceria sexual, número de notificações, óbitos fetais/neonatais atribuíveis à sífilis congênita e taxa de incidência por mil nascidos vivos^6^ ^,^ ^42^ ^-^ ^43^.

A prática também tem previsto espaços locais de discussão dos resultados, com revisão contínua das ações, garantindo a sustentabilidade e adaptação do plano às realidades da rede. A definição clara de prazos e responsabilidades profissionais envolvidos tem possibilitado maior organização e efetividade da proposta. Esse alinhamento entre planejamento e execução, ancorado na vivência dos profissionais nos diferentes pontos da RAS, assegura impacto positivo e duradouro, com gestão mais eficiente dos recursos e maior potencial de transformação no enfrentamento da sífilis congênita^20^.

O estudo apresenta limitações relacionadas à seleção por conveniência, o que pode ter excluído profissionais menos engajados, e ao fato de ter sido realizado em um único município, limitando a generalização dos resultados. A oficina ocorreu em três encontros, conforme o planejado, mas um número maior poderia ter aprofundado as propostas e incluído usuários dos serviços de saúde. Sugere-se que estudos futuros adotem metodologias participativas mais amplas, com maior número de encontros e envolvimento direto dos usuários.

Já as contribuições residem na possibilidade do desenvolvimento de um processo educativo que articula teoria e prática por meio de oficinas participativas. Além disso, demonstra o potencial da práxis como ferramenta de transformação, promovendo a valorização dos saberes locais, o fortalecimento do trabalho em rede e a identificação de fragilidades e potencialidades da RAS. Apontou, ainda, caminhos concretos para superar barreiras como a desarticulação entre serviços, lacunas formativas e baixa adesão a protocolos clínicos. Por fim, a sistematização do plano de ação, com definição de metas, indicadores e atores envolvidos, oferece uma base sólida para monitoramento e avaliação contínua, o que possibilita sua replicação em outros contextos locais, desde que respeitadas as singularidades de cada território.

## Conclusão

A construção participativa com profissionais da RAS de um plano de ações estratégicas para o enfrentamento da SC emergiu como resultado concreto de um processo educativo e transformador, orientado à prática crítica e reflexiva, que promoveu a autonomia e a corresponsabilização dos profissionais. A integração de diferentes olhares e saberes, oriundos da experiência prática e do conhecimento técnico-científico, permitiu a formulação de uma resposta em rede factível, exequível e de custo acessível, contemplando desde o cuidado assistencial até a gestão dos serviços. 

Os encontros evidenciaram o valor da aprendizagem colaborativa e do diálogo horizontal, ao mesmo tempo em que revelaram lacunas na formação e atualização sobre o manejo da SC. Essa constatação reforça a importância da cooperação e do trabalho em rede e da necessidade de ações de educação na saúde, ancoradas no cotidiano das equipes e articuladas às diretrizes clínicas e às necessidades em saúde dos usuários, famílias e comunidades. Destarte, estratégias participativas e integradas, como as aqui desenvolvidas, têm potencial de fortalecer a resposta à SC e de contribuir efetivamente para a melhoria da integralidade do cuidado no SUS.

## Data Availability

Todos os dados gerados ou analisados durante este estudo estão incluídos neste artigo publicado.

# Translation: Construcción participativa de estrategias para el enfrentamiento de la sífilis congénita: investigación-acción^*^

**DOI:** 10.1590/1518-8345.8014.4893

**Authors:** Juliana Sanches Ravagnani, Juliane Andrade, Lucas Cardoso dos Santos, Rúbia Aguiar Alencar

**Affiliations:** 1 Universidade Estadual Paulista, Faculdade de Medicina de Botucatu, Botucatu, SP, Brasil.; 2 Prefeitura de Lins, Secretaria Municipal de Saúde, Lins, SP, Brasil.; 3 Universidade de São Paulo, Escola de Enfermagem, São Paulo, SP, Brasil.

**Keywords:** Sífilis Congénita, Atención a la Salud, Investigación sobre Servicios de Salud, Planificación en Salud, Paquetes de Atención al Paciente, Atención Primaria de Salud.

## Abstract

**Objetivo:**

describir la elaboración de un plan de acción estratégico para combatir la sífilis congénita, realizado de manera participativa con profesionales de la Red de Atención de Salud.

**Método:**

investigación cualitativa, con un enfoque de investigación-acción, basado en el marco freireano. Llevada a cabo en un municipio del interior de São Paulo, contó con la participación de 56 profesionales de la salud. A ausencia de estrategias locales y datos epidemiológicos orientó la realización de un taller en tres reuniones. Las actividades se registraron mediante murales, evaluaciones escritas y diario de campo, analizados mediante Análisis de Contenido.

**Resultados:**

las reuniones promovieron la participación de profesionales, revelaron debilidades en el manejo de la sífilis e impulsaron la formulación de propuestas, como la reestructuración de los procesos de pruebas y diagnóstico, la prescripción y tratamiento oportuno, la búsqueda activa, el seguimiento de los exámenes, el tratamiento de parejas y el manejo adecuado de los casos de sífilis en mujeres embarazadas. Surgieron tres categorías y, como producto final, se preparó el plan de acción estratégico para afrontar la sífilis congénita con aplicabilidad práctica en los servicios de la red.

**Conclusión:**

el proceso colaborativo reforzó la autonomía y la corresponsabilidad de los profesionales, integrando conocimientos técnicos y experiencias locales. El plan se ha implementado y ha contribuido a la cualificación de la respuesta a la sífilis congénita dentro del ámbito del Sistema Unificado de Salud.

## Destacados

**(1)** Plan de acciones estratégicas para el enfrentamiento de la sífilis congénita. **(2)** Investigación-acción como enfoque metodológico que permite la construcción participativa. **(3)** Estrategias participativas e integradas refuerzan el enfrentamiento de la sífilis. **(4)** Importancia de la cooperación y sistematización del trabajo en red. **(5)** Respuesta en red a la sífilis congénita, factible, viable y asequible.

## Introducción 

La sífilis es una infección bacteriana de transmisión sexual, prevenible y curable, que ha sido una preocupación creciente en términos de salud pública global. La Organización Mundial de la Salud (OMS) estima que el número de casos ha aumentado significativamente en los últimos años, con variaciones significativas entre diferentes regiones del mundo^1^ ^-^ ^3^.

Las Américas, con disparidades entre Norteamérica (mayor incidencia), América Latina y el Caribe, concentran la mayor carga global de la enfermedad, con unos 3,37 millones de nuevos casos, lo que corresponde al 42,0% de las infecciones recientes, seguidas por regiones de África y el sudeste asiático^1^ ^,^ ^3^ ^-^ ^4^. Esta tendencia se refleja directamente en el aumento de casos de sífilis congénita (SC), con aproximadamente 68 mil nacimientos vivos afectados al año en todo el mundo^1^ ^,^ ^3^ ^,^ ^5^, con un amplio espectro de manifestaciones clínicas y consecuencias adversas como aborto espontáneo, muerte fetal, prematuridad, bajo peso al nacer, anomalías congénitas y secuelas neurológicas^5^ ^-^ ^8^.

En este contexto, el enfrentamiento de la SC requiere condiciones favorables para el desarrollo de acciones y estrategias destinadas a calificar la atención sanitaria en prenatal y puerperio. Entre ellas, destacan la implementación de políticas de salud pública, el compromiso de los gestores, el trabajo en equipo y la creación de redes, además de una atención prenatal adecuada con captura y vinculación temprana al servicio, pruebas rápidas de sífilis en la primera consulta, al inicio del tercer trimestre y en el momento del parto^6^ ^,^ ^8^ ^-^ ^12^. 

También es esencial un tratamiento oportuno y adecuado con penicilina de benzatina para mujeres embarazadas y sus parejas sexuales, así como el seguimiento postratamiento, la búsqueda activa de ausentes, el registro de los resultados de pruebas y tratamientos en la tarjeta prenatal, y la notificación de casos, entre otros^6^ ^,^ ^8^ ^-^ ^11^. 

Teniendo en cuenta la complejidad de estas intervenciones, se destaca la importancia de las Redes de Atención de Salud (RAS), concebidas como una organización poliárquica de servicios que busca promover la integración sistémica de acciones mediante la provisión de una atención continua, integral y de calidad^10^ ^,^ ^13^.

En este sentido, la creación de redes es esencial para permitir la implementación de estrategias que favorezcan un enfoque integral, la práctica colaborativa interprofesional y la optimización de los flujos de atención, especialmente en el contexto de la atención prenatal en la Atención Primaria de Salud (APS)^10^ ^-^ ^12^, que actúa como centro de coordinación de cuidados y articulador entre los diferentes puntos de las redes^10^ ^,^ ^13^.

Sin embargo, aunque la APS es reconocida como la organizadora de la atención, sigue enfrentando desafíos para coordinar acciones intersectoriales e integradas, esenciales para la prevención de la SC^11^ ^-^ ^13^, como la fragmentación de la atención en las RAS, las desigualdades en el acceso a la salud, el diagnóstico y tratamiento tardíos, el estigma persistente relacionado con las Infecciones de Transmisión Sexual (ITS) y la frágil formación profesional^11^. Además, se observa que muchas estrategias de afrontamiento no se articulan con las realidades locales, lo que compromete la adhesión y eficacia de los planes y políticas implementados^11^ ^,^ ^14^. 

A pesar de estos desafíos, algunas experiencias han demostrado ser exitosas en el enfrentamiento de la SC incorporando intervenciones educativas, iniciativas gubernamentales y programas de mejora^15^ ^-^ ^17^. En este contexto, la enfermería, como la mayor fuerza laboral del sector sanitario y con una presencia significativa en la APS, ocupa una posición estratégica. El trabajo diario de las enfermeras en cuidados prenatales les permite liderar intervenciones orientadas a afrontar la SC^18^ ^-^ ^20^, especialmente en la realización de pruebas rápidas, seguimiento prenatal y seguimiento de casos^18^ ^-^ ^19^. Sin embargo, existen pocos estudios que exploren el papel de la enfermería en la planificación intersectorial y en la elaboración colectiva de estrategias para el enfrentamiento de la SC en redes de salud.

Dada la falta de estudios que integren la construcción colectiva y el fortalecimiento de la red en el enfrentamiento de la SC, este artículo propone contribuir a la construcción de prácticas de atención y gestión en los servicios de salud, basadas en un proceso participativo entre profesionales de la RAS y en la presentación de contenidos teóricos, científicos y prácticos que favorecen el trabajo en red. Así, busca garantizar el derecho a una atención humanizada en el prenatal, el parto y el puerperio, así como la salud del niño. Desde esta perspectiva, se pretende responder a la siguiente pregunta: ¿cómo construir estrategias para el enfrentamiento de la SC en la RAS? Por lo tanto, el objetivo era describir la elaboración de un plan de acción estratégico para afrontar la CS, realizado de manera participativa con profesionales de la RAS.

## Método

### Tipo de estudio

Se desarrolló un estudio cualitativo^21^, basado en el marco teórico freireano^22^, articulado con la metodología de investigación-acción^23^, cumpliendo con las recomendaciones de los *Consolidated Criteria for Reporting Qualitative Research* (COREQ)^24^. 

El marco teórico de Freire, mediante la problematización de la realidad y el diálogo entre sujetos históricos y sociales, sostiene la construcción colectiva del conocimiento. Esta perspectiva se basa en una pedagogía liberadora, orientada a la transformación social, donde la participación activa de los profesionales en la identificación de problemas, en el análisis crítico de prácticas y en la elaboración de soluciones locales dialoga con la concepción de la educación como práctica de la libertad^22^.

Este marco integrado con la metodología de investigación-acción permite la construcción de teorías y caminos que se originan en el propio campo de estudio, que posteriormente se confrontan y validan, con el objetivo de promover cambios significativos ante un problema específico^23^.

Así, se implementa una acción estratégica que requiere la participación activa de los actores implicados en la realidad en cuestión, reconocidos como colaboradores por contribuir conjuntamente a la construcción de todo el proceso, no solo enriqueciendo los resultados finales, sino también promoviendo el compromiso, la pertenencia y soluciones sostenibles^22^ ^-^ ^23^.

La investigación-acción, caracterizada como un proceso continuo de acción-reflexión-acción, se operacionaliza a través de 12 fases que ocurren simultáneamente, de forma flexible y no lineal^23^, como se muestra en la Figura 1.

### Local del estudio

El estudio se desarrolló en un municipio de tamaño medio en el centro-oeste del estado de São Paulo, Brasil, cuya población se estima en 74.779 habitantes^25^, debido a su representatividad dentro de una microrregión de salud compuesta por ocho municipios. La estructura jerárquica y regionalizada de los servicios de salud, con diferentes niveles de complejidad, lo convierte en un lugar estratégico para analizar el funcionamiento del RAS y entender cómo se organizan las políticas y prácticas de atención en contextos reales, especialmente en municipios de tamaño medio.

El municipio del estudio tiene una cobertura del 73,7% de la APS, con una cobertura estimada del 56,1% de la población^26^, con cinco Unidades Básicas de Salud en el modelo tradicional con el Programa de Agentes Comunitarios de Salud y ocho en el modelo de Estrategia Salud de la Familia. También cuenta con un equipo multiprofesional (eMulti) y servicios de atención especializada, como clínicas ambulatorias de ITS y prenatales de alto riesgo, así como un hospital de maternidad. La gestión de servicios de la APS y de complejidad media la realiza una *Organização Social de Saúde* (“organización social de salud”) y la de alta complejidad por una institución filantrópica.

El municipio de estudio presenta una serie de retos que motivaron la realización de la investigación, tales como la notificación insuficiente de casos, tratamientos inadecuados o no realizados, diagnósticos tardíos, menos de seis consultas prenatales y resultados desfavorables como abortos, mortinatalidad y mortalidad infantil, lo que puso de manifiesto la fragilidad de la atención prestada a las mujeres embarazadas y a los recién nacidos.

### Periodo de la recogida de datos

La recogida de datos se realizó entre abril y agosto de 2023.

### Participantes

Los colaboradores de la investigación comprendieron a todos los profesionales de la salud con formación superior insertados en la RAS de este municipio, sumando un total de 80 colaboradores, a saber: trabajadores sociales, enfermeros y médicos que trabajan en la APS, servicios de media y alta complexidad; nutricionista, psicóloga y profesional de educación física de la eMulti; miembros del Comité de Mortalidad Materna, Infantil y Fetal; gestores de la *Secretaria Municipal de Saúde* (“Departamento Municipal de Salud”) que trabajan en los departamentos de Vigilancia Epidemiológica, Salud Colectiva, Evaluación y Control. Además, en vista de la representatividad en la RAS, había un consejero/enfermero del *Conselho Regional de Enfermagem* (“Consejo Regional de Enfermería”) de São Paulo y una enfermera que articulaba entre la APS y la *Diretoria Regional de Saúde* (“Junta Regional de Salud”).

### Criterios de selección y definición de los participantes 

El criterio de selección de los colaboradores fue por conveniencia. La invitación se hizo a los 80 profesionales elegibles para el estudio, considerando como criterios de inclusión la presencia de un profesional de la salud con educación superior, que realice atención directa y/o indirecta a personas con SG y SC. Como criterios de exclusión durante el periodo de recogida de datos, se consideraron personas de baja médica o vacaciones.

Para garantizar una amplia participación de profesionales en el estudio, se envió una invitación por WhatsApp con información sobre el objetivo de la investigación, justificación y un enlace para la preinscripción en Google *Forms*, ofreciendo opciones para participar en turnos de mañana o tarde para no comprometer la atención en la RAS. Al mismo tiempo, la *Secretaria Municipal de Saúde* emitió un documento circular reforzando la importancia de la participación. Tras identificar a los interesados por el formulario, hubo un contacto telefónico con quienes no estaban registrados para la concienciación. Al finalizar la preinscripción, 56 profesionales confirmaron su interés. Después, se creó una lista de transmisión por WhatsApp para confirmar la participación, transmitir fechas y horas, y enviar un nuevo enlace para validar el registro, vinculado a la Sección Técnica responsable de emitir los certificados.

### Instrumentos de recogida de datos

Los instrumentos utilizados para registrar las informaciones fueron: cuestionario sociodemográfico para caracterizar a los colaboradores, muro de palabras elaborado en dinámicas colectivas, muro de comentarios desde el punto de vista de los colaboradores, evaluaciones aplicadas al final de cada reunión y el diario de campo. Todas estas formas de recogida de datos fueron registradas por escrito por los colaboradores. Cabe destacar que el diario de campo para registrar el contexto colectivo, en el momento de los talleres, fue observado por un psicólogo formado por los tres investigadores que recopilaron los otros datos. Los instrumentos no han sido probados previamente. 

Las evaluaciones y el cuestionario sociodemográfico se realizaron mediante Google *Forms*, disponibles en código QR e impresos en caso de que el colaborador no tuviera acceso a él. Para el mural de palabras y las evaluaciones, se utilizó la aplicación Wordle, capaz de generar nubes de palabras, lo que permite visualizar de forma destacada las palabras que aparecen con más frecuencia en el texto, expresando de forma sintética la idea del grupo en su conjunto.

Para la elaboración del plan de acción, se utilizó una hoja de cálculo, caracterizada como instrumento de planificación, que contenía la identificación de la acción, potencialidades, debilidades y propuesta de intervención que posteriormente culminaba en el instrumento final con la siguiente información: objetivos, acciones, planificación, servicio y categoría de profesional responsable.

### Recogida de datos

La recogida de datos siguió a las 12 fases de la investigación-acción (Figura 1), con un taller basado en la relación entre el problema de la sífilis y la realidad práctica, lo que permitió la construcción de una intervención estratégica dirigida a las necesidades identificadas en la realidad del trabajo de los colaboradores.

**Figura 1:** Detalles de las fases de la investigación-acción sobre la construcción de estrategias para el enfrentamiento de la sífilis congénita

Fases de la investigación-acción	Teorización	Desarrollo
**I** **Fase exploratoria**	Levantamiento de datos a investigar, enumerando los problemas emergentes y las acciones que deben llevarse a cabo.	Revisión integrativa y diagnóstico situacional basados en datos epidemiológicos sobre sífilis en mujeres embarazadas y sífilis congénita.
**II** **Tema de la investigación**	Basándose en la exposición del problema práctico a abordar/estudiar.	Reuniones con profesionales exponiendo el problema y consensando sobre la construcción de la estrategia.
**III** **Colocación de los problemas**	Definición de los principales problemas identificados en la fase exploratoria.	Las reuniones permitieron la exposición del problema y la concienciación para la toma de decisiones.
**IV** **El lugar de la teoría**	Base teórica/científica, que consiste en la articulación del problema con un marco teórico adaptado a la investigación.	A partir del marco teórico de Freire se desarrolló un proceso dialógico y emancipatorio, en el que los sujetos reflexionaban críticamente sobre su realidad.
**V** **Hipótesis**	La suposición formulada por el investigador impregna el mantenimiento del enfoque en el tema, buscando las soluciones necesarias para afrontar el problema.	Los colaboradores fueron llevados a la reflexión, lo que permitió analizar acciones, potencialidades y debilidades encontradas en los servicios.
**VI** **Seminarios**	Momento en el que la información será recopilada, analizada y sometida a interpretaciones, estableciendo directrices para la acción que se pondrán a prueba en la práctica.	Reuniones presenciales entre investigadores y colaboradores, llamadas talleres, impregnando las fases de la investigación-acción.
**VII** **Campos de observación, muestreo**	Delimitación del campo de observación según su relevancia para un sujeto dado.	Los colaboradores fueron intencionalmente seleccionados según su implicación con el tema en sus áreas de actuación.
**VIII** **Recogida de datos**	Se caracteriza por la producción de material para análisis y dirección de la investigación.	Levantamiento y tratamiento de los datos epidemiológicos y contenido producido en el taller de trabajo.
**IX** **Aprendizaje**	Asociado al proceso de investigación, el conocimiento teórico, la producción de conocimientos y la elaboración de propuestas.	Aprendizaje simultáneo entre investigadores y colaboradores a lo largo del periodo de investigación.
**X** **Saber formal e informal**	Búsqueda de conocimientos mediante estructuras de comunicación entre las dimensiones de las distintas fases de la investigación.	La construcción de conocimientos formales e informales se llevó a cabo en todas las fases del desarrollo del taller.
**XI** **Plan de acción**	Los miembros participan en debates que permiten diseñar una acción que resuelva un problema.	Construcción de un plan de acción para combatir la sífilis congénita, que incluya objetivos, ejecución, sectores y profesionales implicados.
**XII** **Divulgación externa**	La retroalimentación sobre los resultados obtenidos en la investigación debe ser accesible y comprensible para todas las personas.	El plan de acción fue posteriormente entregado a la dirección municipal y a toda la Red de Atención de Salud.

El proceso se inició a partir de una revisión integradora de la literatura, que evidenció una carencia de conocimientos relacionados con las estrategias prenatales e intervenciones destinadas a afrontar la SC. Este movimiento correspondió a las fases I - Fase exploratoria, IV - El lugar de la teoría, IX - Aprendizaje y X - Conocimiento formal e informal. Al mismo tiempo, se recogieron datos epidemiológicos de las notificaciones de SG y SC, permitiendo un diagnóstico situacional del tema, de acuerdo con las fases I - Fase exploratoria, III - Colocación del problema y VIII - Recogida de datos.

El taller de trabajo se estructuraba mediante la planificación con apoyo didáctico y pedagógico, que incluía tiempo, duración, empleados, propuestas, objetivos, desarrollo, recursos utilizados y productos esperados. Su operacionalización se llevó a cabo mediante tres reuniones presenciales semanales, con una duración media de tres horas y media cada una. La actividad se ofreció a dos clases diferentes, por la mañana y la tarde, lo que favorecía una mayor fidelidad de los empleados, sin comprometer la continuidad de los servicios de atención. Esta etapa correspondió a las fases III - Colocación de problemas, IV - El lugar de la teoría, V - Hipótesis, VI - Seminarios, VIII - Recogida de datos, IX - Aprendizaje, X - Conocimiento formal e informal y XI - Plan de acción.

En resumen, la primera reunión del taller buscó presentar la propuesta de trabajo, la construcción del flujo de atención para mujeres embarazadas habituales y mujeres embarazadas con sífilis en la RAS, y la colocación del problema con el diagnóstico situacional mediante datos epidemiológicos. La segunda reunión abordó la teoría dialogada con base científica e inmersión en documentos técnicos y protocolo gubernamental de orientación, casos que desencadenan la SC con evolución a SG con la identificación de la acción, potencialidades y debilidades observadas en el caso. Finalmente, en la tercera reunión se presentaron las impresiones generadas por las discusiones de los casos, la importancia de la notificación obligatoria y la consolidación de la planificación, que contenían objetivos, acciones, calendario, categoría profesional y servicio responsable, lo que permitió la construcción del producto final por ambas clases (mañana y tarde).

### Análisis de datos

Los datos cuantitativos obtenidos mediante el cuestionario sociodemográfico, utilizados para caracterizar a los colaboradores, se analizaron basándose en técnicas estadísticas. Por otro lado, la información del muro de palabras, elaborada en dinámica colectiva, el muro de comentarios, las evaluaciones realizadas al final de cada reunión y el diario de campo se organizaron e interpretaron mediante la técnica de análisis de contenidos, propuesta por Bardin, adaptada para investigación guiada por la metodología de investigación-acción^27^ ^-^ ^28^.

El análisis de contenido comenzó con la lectura flotante de todo el material generado en el taller, seguida de la formulación de hipótesis provisionales y la transformación de la mayor parte del texto en una unidad de registro, asociada a temas/unidades de significado. Así, cada tema/unidad de significado estaba compuesto por un conjunto de unidades de registro. Basándose en la determinación y cuantificación de los temas/unidades de significado, se definieron las dimensiones de emergencia, agrupándolas según las hipótesis de análisis, conformando la construcción de categorías^27^ ^-^ ^28^.

Para garantizar la confidencialidad de los colaboradores, se asignó un código alfanumérico compuesto por la letra “C” seguida de un dígito para presentar extractos del análisis de contenido del taller de trabajo, el símbolo [...] se consideró cuando se omitió alguna parte.

Los datos se analizaron a la luz del marco teórico de Freire^22^, que valora la participación activa de los individuos en la construcción del conocimiento, animándoles a reflexionar sobre su realidad y transformarla mediante acciones colectivas y comprometidas.

### Consideraciones éticas

Los colaboradores recibieron el Formulario de Consentimiento Informado con orientación sobre los objetivos de la investigación y la participación voluntaria. Se garantizó el anonimato, la confidencialidad y la libertad de retirarse en cualquier momento y sin prejuicio. Las actividades se llevaron a cabo en entornos acogedores, con escucha activa y respeto, con el objetivo de evitar cualquier incomodidad o vergüenza. Aprobación obtenida por el Comité de Ética de la Investigación bajo el Dictamen n.º 5.977.502/CAAE 66686923.7.0000.5411 el 31/03/2023.

## Resultados

Un total de 56 profesionales de la salud (70,0% de los invitados) participaron en esta investigación, en su mayoría mujeres (46) y cisgénero (55), con una edad media de 37,5 años. La mayoría estaban casados o en una unión estable (33).

En cuanto a la formación, participaron 33 enfermeros, 12 médicos, ocho trabajadores sociales, un psicólogo, un nutricionista y un profesional de educación física. De estos, 22 tenían especialización, tres tenían un máster y uno un doctorado; 16 ocuparon cargos directivos. La duración del tiempo en el campo osciló entre 6 y 30 años (de media 9). En cuanto a la sífilis, 33 habían participado en formación, 29 estaban cualificados para pruebas rápidas y 36 se consideraban capaces de manejar la sífilis en mujeres embarazadas (SG) y 34 de la SC.

El análisis de datos generado en las reuniones del taller de trabajo resultó en tres categorías: Conociendo y sensibilizando al grupo; Fundamentación teórica y aplicabilidad en la realidad; Construyendo estrategias para el enfrentamiento de la sífilis congénita (Figura 2).

**Figura 2:** Síntesis de la síntesis de categorías sobre la construcción de estrategias para el enfrentamiento de la sífilis congénita

Temas/ Unidades de Significación	Unidad de Registro/ Tema n (%)	Categoría	Unidad de Registro/ Categoría n (%)
Relevancia de la temática, oportunidad de aprendizaje e intercambio de experiencias, sensación de pertenencia y satisfacción al participar en este taller.	226 (29,2)	“Conociendo y sensibilizando el grupo”	286 (37,0)
La presencia de profesionales de la salud que forman la Red de Atención de Salud, la importancia del trabajo en equipo y la valorización de las reuniones participativas.	60 (7,8)		
Deficiencia de conocimiento sobre la sífilis e inseguridad en el cuidado de la sífilis en mujeres embarazadas y sífilis congénita, identificando debilidades y divergencias en el cumplimiento de protocolos y conductas.	68 (8,8)	“Fundamentación teórica y aplicabilidad en la realidad”	136 (17,6)
Compreendendo a Rede de Atenção à Saúde e refletindo sobre a complexidade do cuidado às gestantes com sífilis. Comprendiendo la Red de Atención de Salud y reflexionando sobre la complejidad de la atención a las mujeres embarazadas con sífilis.	32 (4,1)		
Conociendo los datos epidemiológicos sobre casos de sífilis en mujeres embarazadas y de sífilis congénitas, divulgación periódica a la Red de Atención de Salud.	36 (4,7)		
Implementando el desarrollo de estrategias para el enfrentamiento de la sífilis en mujeres embarazadas y de sífilis congénitas, con el objetivo de cambiar la práctica laboral.	98 (12,7)	“Construyendo estrategias para el enfrentamiento de la Sífilis Congénita”	350 (45,4)
Organizar más reuniones en el taller, desarrollar otros eventos que aborden la temática, ampliación a otras categorías profesionales de la Red de Atención de Salud, creación de un grupo de trabajo permanente sobre sífilis en mujeres embarazadas y congénitas.	77 (10,0)		
Satisfacción al colaborar en la elaboración de un plan, seguridad con el inicio de nuevas perspectivas de cambio y reflexiones en relación con la atención de mujeres embarazadas con sífilis.	175 (22,7)		
Total	772 (100,0)		

La categoría “Conociendo y sensibilizando al grupo” contemplaba el proceso de acogida, escucha y construcción colectiva, revelando la valorización del espacio de intercambio, la movilización subjetiva de profesionales y el sentimiento de pertenencia a la propuesta. Destacan la adhesión activa a la propuesta de trabajo, el reconocimiento de la importancia del tema y la implicación en las actividades del taller: *Estamos teniendo la oportunidad de intercambiar experiencias e ideas para mejorar la calidad del servicio* [...] (C25); [...] *es bueno que haya espacio para el diálogo y compartir incluso experiencias que dificultan la obra* (C40)*.*

La implicación de profesionales que componen los diferentes puntos de la RAS se percibió como un elemento potenciador para la reflexión y la propuesta de acciones y estrategias en equipo: *Toda la red participa en el taller, ya que el conocimiento de cada uno aporta y aporta la información necesaria para mejorar el servicio* [...] (C27); *Hubo adhesión por parte de varios segmentos y profesionales dentro del sector sanitario, ya que esto enriquece el taller y aporta más experiencias y experiencia a todos* [...] (C40)*.*

La categoría “Fundamentación teórica y aplicabilidad en la realidad” comprendía la articulación teórica con la realidad experimentada por los colaboradores del taller que relataron lagunas en el conocimiento sobre sífilis, inseguridad en la gestión y divergencias en las prácticas adoptadas entre los puntos de la RAS, concienciando sobre la necesidad de cambios: *Que “en la” red de salud existen divergencias y diferencias en la atención de mujeres embarazadas con sífilis* (C25); *No contamos con todos los recursos o protocolos que se cumplen en nuestro municipio* (C23).

El análisis de las prácticas y el funcionamiento del trabajo en red nos permitió reconocer la complejidad de la atención para las mujeres embarazadas con sífilis y la importancia de flujos de atención bien definidos: *Hay un protocolo y un flujo que debe seguirse para que no haya inconsistencias ni riesgos* [...] (C49); *Que si hubiera un consenso entre médicos y enfermeros respecto a la autonomía de la prescripción* [...] (C19)*.*

El compartimiento de datos epidemiológicos locales contribuyó a una conciencia crítica de la realidad del territorio, subrayando la relevancia de difundir periódicamente estos datos a los servicios de la RAS: *la información estadística “sirvió” como base para reflejar nuestras acciones* (C25); [...] *es una pena que esta tasa siga siendo muy alta, ya que es una enfermedad totalmente prevenible* (C21)*.*

La categoría “Construyendo estrategias para el enfrentamiento de la sífilis congénita” abarcó la eficacia en la construcción participativa de estrategias, basadas en reflexiones colectivas, que permiten la producción de una poderosa herramienta de gestión, capaz de transformar la realidad práctica.

Los colaboradores expresaron el deseo de transformar las reflexiones y el aprendizaje en acciones prácticas: *podríamos convertir este aprendizaje en una rutina de atención estandarizada* (C15)*; Elaborar un protocolo municipal, de prevención y tratamiento* (C23); *Si creas un protocolo o seguimiento específico, todos deberían trabajar de la misma manera* (C26)*.*

También surgió la propuesta de crear reuniones periódicas que abordaran el tema con la participación de otros profesionales que trabajan en la RAS, además de la creación de un grupo de trabajo permanente sobre SG y SC: *crear un grupo de trabajo permanente* (C2); [...] *realizar reuniones periódicas para el análisis de datos en el municipio y la elaboración de ajustes en estrategias cuando sea necesario* (C24).

La satisfacción con el proceso de construcción, el sentimiento de corresponsabilidad y el inicio de cambios en relación con el cuidado de mujeres embarazadas con sífilis eran evidentes: *se fortaleció la red, mejorando la comunicación entre sectores y entre el propio equipo, todos “hablando el mismo idioma”* [...] (C27); *Será estupendo lograr el objetivo del taller y, en unos años, cosechar los frutos de estas reuniones con la reducción de casos de sífilis congénita* (C48); [...] *Mostrar la realidad nos hace pensar, reflexionar y ver situaciones con las que no tendríamos contacto si no existieran tales reuniones* (C55).

El taller de trabajo proporcionó a los colaboradores conocimientos, conciencia, fundamentos teóricos y la elaboración de un plan de acción estratégico para afrontar la informática, caracterizado como un producto técnico/científico, detallado en 19 objetivos, seis de los cuales están dirigidos a todas las mujeres embarazadas y 13 a mujeres embarazadas con sífilis. Cada objetivo se detalla en un conjunto de acciones, con un calendario, descripción de los servicios y categorías profesionales implicadas, constituyendo un instrumento para su aplicación en la práctica.

Los objetivos del plan de acción para todas las mujeres embarazadas eran: identificar el embarazo temprano para el inicio oportuno de la atención prenatal (antes de las 12 semanas); realizar consultas de enfermería prenatal en todas las unidades de salud; realizar pruebas rápidas de VIH, sífilis y hepatitis B y C en la primera visita prenatal; repetir pruebas rápidas en un plazo de 30 días en una situación de exposición en los últimos tres meses; realizar pruebas rápidas en el segundo y tercer trimestre y en el momento del parto; y realizar la atención prenatal de la asociación.

Los objetivos dirigidos a las mujeres embarazadas con sífilis incluían: diagnosticar, prescribir y tratar la sífilis de manera oportuna según la fase clínica; solicitar una prueba no treponémica del *Venereal Disease Research Laboratory* (VDRL) con recogida de muestras en el momento del diagnóstico; notificar simultáneamente con el diagnóstico; identificar casos de cicatrices sorológicas de sífilis; cuidar la relación de la mujer embarazada; notificar a la pareja si se ha confirmado la sífilis; registrar y supervisar la administración de penicilina en mujeres embarazadas y parejas; recoger y monitorizar la prueba mensual de VDRL para curar o controlar la reinfección; realizar una búsqueda activa de mujeres embarazadas ausentes y parejas; mantener actualizado el historial de tratamiento y el seguimiento de la prueba VDRL en mujeres embarazadas con sífilis con los servicios interesados; definir el flujo de laboratorio respecto a la correcta finalización de la solicitud de prueba y el cumplimiento del diagrama de flujo diagnóstico de sífilis (enfoque inverso); interpretar correctamente el diagrama de flujo diagnóstico de sífilis, en relación con el uso de la prueba confirmatoria treponémica; y asegurar el manejo adecuado de las mujeres embarazadas con sífilis y del recién nacido en el momento del parto.

El aplazamiento de la ejecución de las acciones se planificó en función de parámetros a corto, medio y largo plazo. Considerando la implementación como el principio, las acciones a corto plazo se refieren a las demandas más urgentes, fáciles de implementar, que se espera que ocurran en los primeros tres meses de la implementación; Las acciones a medio plazo cubren actividades que requieren más tiempo para su organización y desarrollo, con ejecuciones esperadas en hasta seis meses; Las acciones a largo plazo, en cambio, requieren más recursos, continuidad y tiempo, y están programadas para durar hasta 12 meses.

Los servicios implicados en el desarrollo de las acciones estuvieron representados por las áreas de atención de la APS y la gestión de los departamentos de *Saúde Coletiva*, *Vigilância Epidemiológica e Programa Municipal de IST/Aids/Hepatites Virais* (“Salud Colectiva, Vigilancia Epidemiológica y Programa Municipal de ETS/SIDA/Hepatitis Viral”). Las categorías profesionales implicaban: agentes de salud comunitaria, enfermeros, médicos, profesionales de eMulti y gestores de los servicios de la RAS.

## Discusión 

La propuesta para la construcción participativa de estrategias para el enfrentamiento de la SC a la luz de la investigación-acción y basada en el marco teórico de Freire permitió un proceso educativo y transformador entre investigadores y colaboradores. Este proceso implicó la identificación de desafíos, la problematización de la realidad y la construcción colectiva de soluciones mediante la articulación entre teoría y práctica, lo que permitió esbozar un plan de acción para lograr cambios en la perspectiva del trabajo compartido por la RAS respecto al enfrentamiento de la SC.

El taller de trabajo proporcionó un espacio para el diálogo horizontal que articulaba el conocimiento técnico-científico y el conocimiento de la práctica laboral, favoreciendo el intercambio de experiencias entre diferentes categorías profesionales y puntos de la red, con el fin de promover el aprendizaje mutuo y la reflexión colectiva. Cuando diferentes categorías profesionales exponen su punto de vista en un ejercicio de diálogo, combinado con la literatura científica y la experiencia de los sujetos, se moviliza la elaboración de concepciones e ideas críticas^29^ ^-^ ^30^, capaces de transformar la realidad a través de la praxis.

La problematización de la realidad local basada en datos epidemiológicos, combinada con la experiencia concreta de los colaboradores, permitió activar la conciencia crítica. Este movimiento de sensibilización permitió reconocer las debilidades de la red, como la desarticulación de servicios, el bajo cumplimiento de protocolos y la falta de conocimiento de documentos técnicos, así como potencialidades locales, como el trabajo intersectorial y el compromiso de los profesionales.

La discusión también reveló una tensión entre la percepción de competencia de los profesionales y los datos epidemiológicos y normativos. Algunos de los colaboradores informaron sentirse preparados para la gestión de la SC, aunque los indicadores locales y los informes de conducta inapropiada indican lo contrario. Esta contradicción puede reflejar carencias en la formación, conocimientos obsoletos o incluso la naturalización de prácticas erróneas en las que la percepción de suficiencia no garantiza la adhesión a conductas basadas en la evidencia, lo que hace necesario fomentar la reflexión crítica sobre la propia práctica^20^ ^,^ ^31^.

Un manejo adecuado de la infección por sífilis durante el embarazo es esencial para reducir las complicaciones maternas y neonatales, así como para prevenir la transmisión vertical al feto. Por ello, los profesionales de la salud deben estar informados sobre el diagnóstico, tratamiento y seguimiento de la SC según las directrices nacionales e internacionales^6^ ^,^ ^15^ ^,^ ^19^ ^,^ ^31^. Sin embargo, un número considerable de colaboradores informó que no se sentían capaces de gestionar la SG y la SC, así como no conocían los documentos técnicos que guiaban el cuidado/manejo de esta enfermedad y los flujos de atención entre los diferentes puntos de la RAS.

La dificultad de acceder a documentos y protocolos técnicos puede representar una barrera crítica en la organización del trabajo de los equipos. Protocolos desactualizados, no compartidos, con lenguaje inaccesible o no incorporados a la rutina de los servicios son obstáculos relevantes. Los estudios destacan que la incorporación de tecnologías educativas en la práctica favorece la adhesión y actualización de los equipos^16^ ^-^ ^17^ ^,^ ^19^ ^,^ ^31^, además de la adopción de tecnologías digitales simples, como códigos QR, sistemas informatizados integrados con flujos de atención, materiales visuales adaptados y talleres periódicos basados en protocolos actualizados.

La inconsistencia en la aplicación de protocolos clínicos pone de manifiesto problemas recurrentes en los servicios de salud. Cuando las directrices no se difunden o comprenden bien, pueden producirse fallos en el diagnóstico y el tratamiento, con un consecuente impacto negativo en la atención en salud^17^ ^,^ ^31^ ^-^ ^32^. Además, la fragilidad en el cumplimiento de protocolos y las divergencias en las conductas evidencian la necesidad de estandarización y refuerzo en la difusión de guías y protocolos clínicos, lo que promueve la mejora y el rendimiento de los profesionales, así como fortalece la confianza de la población en el sistema de salud, favoreciendo mejores resultados clínicos y una atención más segura y cualificada^17^ ^,^ ^31^ ^-^ ^32^.

Para superar esta fragilidad, se recomienda invertir en acciones de formación continua y permanente basadas en metodologías activas^33^ ^-^ ^34^, la difusión de materiales educativos simplificados y accesibles, en formatos impresos y digitales^35^ ^-^ ^36^ y el fortalecimiento del trabajo en red, promoviendo debates basados en casos reales^37^ ^-^ ^39^. La sistematización de conductas permite a todos los profesionales seguir recomendaciones basadas en la evidencia, lo que minimiza las variaciones en la atención y garantiza la seguridad en la atención proporcionada.

En este contexto, los talleres participativos expresados en el ciclo continuo de acción-reflexión-acción son iniciativas que refuerzan el diálogo y proporcionan una base sólida para la adquisición de conocimientos técnico-científicos para estandarizar las prácticas y mejorar la calidad de la atención^33^ ^-^ ^34^ ^,^ ^40^ mediante discusiones colaborativas, resolución de problemas y una mayor implicación en el trabajo en salud^20^ ^,^ ^37^ ^,^ ^39^ ^,^ ^41^.

Así, la toma de decisiones asertiva frente a una propuesta realista integra el trabajo en equipo y la red, y fortalece la comunicación, alineando las acciones entre diferentes servicios^37^ ^-^ ^39^ ^,^ ^41^, colaborando para mejorar la calidad de las acciones propuestas, así como la facilidad de implementación y monitorización.

El plan de acción está en proceso de implementación concreta, acompañado de un seguimiento sistemático y evaluaciones continuas. La eficacia de este proceso ya empieza a hacerse evidente en la rutina de los servicios, con el uso de indicadores cuantitativos y cualitativos que permiten medir la adherencia, eficacia e impacto de las acciones desarrolladas. Entre los principales indicadores adoptados, destacan los siguientes: pruebas oportunas (1º, tercer trimestre y parto), tiempo entre el diagnóstico y el inicio del tratamiento, proporción de mujeres embarazadas tratadas con el régimen adecuado de penicilina con benzatina, tratamiento de las parejas sexuales, número de notificaciones, muertes fetales/neonatales atribuibles a sífilis congénita y tasa de incidencia por cada mil nacidos vivos^6^ ^,^ ^42^ ^-^ ^43^.

La práctica también ha proporcionado espacios locales para debatir los resultados, con una revisión continua de las acciones, asegurando la sostenibilidad y adaptación del plan a las realidades de la red. La definición clara de plazos y responsabilidades profesionales implicadas ha permitido una mayor organización y eficacia de la propuesta. Esta alineación entre planificación y ejecución, basada en la experiencia de profesionales en diferentes puntos de la RAS, garantiza un impacto positivo y duradero, con una gestión más eficiente de los recursos y un mayor potencial de transformación en el enfrentamiento de la sífilis congénita^20^.

El estudio presenta limitaciones relacionadas con la selección por conveniencia, que puede haber excluido a profesionales menos comprometidos, y el hecho de que se llevó a cabo en un solo municipio, lo que limita la generalización de los resultados. El taller se llevó a cabo en tres reuniones, como estaba previsto, pero un número mayor podría haber profundizado en las propuestas e incluido a los usuarios de servicios sanitarios. Se sugiere que futuros estudios adopten metodologías participativas más amplias, con un mayor número de reuniones y una mayor participación directa de los usuarios.

Las contribuciones, por otro lado, residen en la posibilidad de desarrollar un proceso educativo que articule teoría y práctica a través de talleres participativos. Además, demuestra el potencial de la praxis como herramienta de transformación, promoviendo la apreciación del conocimiento local, el fortalecimiento de la red y la identificación de debilidades y potencialidades de la RAS. También señaló formas concretas de superar barreras como la desarticulación entre servicios, las brechas en la formación y el bajo cumplimiento de protocolos clínicos. Finalmente, la sistematización del plan de acción, con la definición de objetivos, indicadores y actores implicados, ofrece una base sólida para un seguimiento y evaluación continuos, lo que permite su replicación en otros contextos locales, siempre que se respeten las singularidades de cada territorio.

## Conclusión

La construcción participativa con profesionales de la RAS de un plan de acción estratégico para el enfrentamiento de la SC surgió como resultado concreto de un proceso educativo y transformador, orientado a la práctica crítica y reflexiva, que promovió la autonomía y la corresponsabilidad de los profesionales. La integración de diferentes perspectivas y conocimientos, surgidos de la experiencia práctica y el conocimiento técnico-científico, permitió la formulación de una respuesta de red factible, viable y asequible, que abarca desde la atención hasta la gestión de servicios.

Las reuniones destacaron el valor del aprendizaje colaborativo y el diálogo horizontal, al tiempo que se revelaron lagunas en la formación y la actualización sobre la gestión de la SC. Este hallazgo refuerza la importancia de la cooperación y del trabajo en rede y de la necesidad de acciones de educación en salud, ancladas en la vida diaria de los equipos y articuladas con las directrices clínicas y las necesidades de salud de los usuarios, familias y comunidades. Así, las estrategias participativas e integradas, como las desarrolladas aquí, tienen el potencial de fortalecer la respuesta a la SC y contribuir eficazmente a la mejora de la integralidad de la atención en el SUS.

## Data Availability

Todos los datos generados o analizados durante este estudio están incluidos en este artículo publicado.
